# Multi-gene panel testing in Korean patients with common genetic generalized epilepsy syndromes

**DOI:** 10.1371/journal.pone.0199321

**Published:** 2018-06-20

**Authors:** Cha Gon Lee, Jeehun Lee, Munhyang Lee

**Affiliations:** 1 Department of Pediatrics, Nowon Eulji Medical Center, Eulji University, Seoul, Republic of Korea; 2 Department of Pediatrics, Samsung Medical Center, Sungkyunkwan University School of Medicine, Seoul, Republic of Korea; University of Catanzaro, ITALY

## Abstract

Genetic heterogeneity of common genetic generalized epilepsy syndromes is frequently considered. The present study conducted a focused analysis of potential candidate or susceptibility genes for common genetic generalized epilepsy syndromes using multi-gene panel testing with next-generation sequencing. This study included patients with juvenile myoclonic epilepsy, juvenile absence epilepsy, and epilepsy with generalized tonic-clonic seizures alone. We identified pathogenic variants according to the American College of Medical Genetics and Genomics guidelines and identified susceptibility variants using case-control association analyses and family analyses for familial cases. A total of 57 patients were enrolled, including 51 sporadic cases and 6 familial cases. Twenty-two pathogenic and likely pathogenic variants of 16 different genes were identified. *CACNA1H* was the most frequently observed single gene. Variants of voltage-gated Ca^2+^ channel genes, including *CACNA1A*, *CACNA1G*, and *CACNA1H* were observed in 32% of variants (*n* = 7/22). Analyses to identify susceptibility variants using case-control association analysis indicated that *KCNMA1* c.400G>C was associated with common genetic generalized epilepsy syndromes. Only 1 family (family A) exhibited a candidate pathogenic variant p.(Arg788His) on *CACNA1H*, as determined via family analyses. This study identified candidate genetic variants in about a quarter of patients (*n* = 16/57) and an average of 2.8 variants was identified in each patient. The results reinforced the polygenic disorder with very high locus and allelic heterogeneity of common GGE syndromes. Further, voltage-gated Ca^2+^ channels are suggested as important contributors to common genetic generalized epilepsy syndromes. This study extends our comprehensive understanding of common genetic generalized epilepsy syndromes.

## Introduction

A proposal written by the 2010 International League Against Epilepsy (ILAE) recommends changing the term “idiopathic” to “genetic” when describing epilepsy syndromes [1]. Idiopathic/genetic generalized epilepsy (IGE/CGE) is classically categorized into several commonly recognizable sub-syndromes. Juvenile myoclonic epilepsy (JME), childhood absence epilepsy (CAE), juvenile absence epilepsy (JAE), and epilepsy with generalized tonic-clonic seizures alone (EGTCS) are the most common GGE subtypes [2, 3]. Of the common GGE sub-syndromes, JME, JAE, and EGTCS (but not CAE) are lifelong diseases with overlapping clinical features.

As demonstrated in family and twin studies, genetic factors are major contributors to common GGE syndromes, with heritability estimates as high as 80% in monozygotic twins [4–6]. Two different heritability models have been used to explain the genetic basis of common GGE syndromes, including Mendelian inheritance of a few major genes or simultaneous involvement of multiple genes with minor effects inherited in a non-Mendelian fashion [7]. Several GGE causative or susceptibility genes have been identified using linkage analyses of Mendelian GGE inheritance patterns and dozens of susceptibility alleles that have been reported following association analyses of non-Mendelian GGE inheritance patterns [7–9]. Despite the many findings associated with genetic studies of common GGE syndromes, a comprehensive understanding of these findings is limited by high genetic complexity and heterogeneity [2, 10]. A multi-gene panel testing with next-generation sequencing is more useful for analyzing diseases with high genetic heterogeneity than broader approaches such as whole exome sequencing and whole genome sequencing. Focusing on important genes or regions facilitates detailed sequencing, thus enabling rare variants to be identified [11].

Literature reviews support the present study design, which includes an epilepsy multi-gene panel testing for potential candidate or susceptibility genes. The study aimed to investigate associations between these genes and common GGE syndromes, including JME, JAE, and EGTCS. Furthermore, we focused on identifying causative and susceptibility genes with causative and susceptibility variants or genes using a customized multi-gene panel testing.

## Materials and methods

### Participant inclusion and exclusion criteria

A cross-sectional retrospective chart review was conducted for all patients with epilepsy who were clinically diagnosed with a common GGE syndrome (JAE, JME, or EGTCS). All patients were monitored and followed up at Samsung Medical Center or Eulji General Hospital from 1990 to 2015. The diagnosis of epilepsy was made by pediatric neurologists. Epilepsy syndromes were strictly diagnosed based on the clinical and electroencephalography features proposed by the ILAE [3]. There was no age limit for study enrollment. Patients with a history of major psychiatric disorders (autism spectrum disorder, schizophrenia, affective disorders, or recurrent episodes requiring pharmacotherapy or treatment in a hospital) or intellectual disability were excluded.

### Ethics statement

Ethical approval for this retrospective study was provided by the institutional review board of Samsung Medical Center (2014-07-001-004) and the ethical committee of Nowon Eulji Medical Center (2014-06-015-001). Written informed consent for genetic testing was obtained from all participants before the study began. Written informed consent was obtained from the parents for child participants; the child's assent was also obtained.

### Control group

The Korean Reference Genome Database (KRGDB) houses a publicly available population web browser containing Ansan-Ansung cohort data from the Korean Genome and Epidemiology Study. In the present study, control group were from public genetic databases of 1100 Korean individuals from the Ansan-Ansung cohorts. The whole genome of 1100 Korean individuals were sequenced using an Illumina Hiseq2000 sequencer. The raw sequences were mapped to reference genome hg19 from the University of California Santa Cruz (original GRCh37 from the National Center for Biotechnology Information, Feb. 2009) via the Burrows-Wheeler alignment tool, and the mapped sequences were analyzed using sequence alignment map (SAM) tools. To ensure sequencing accuracy, the average sequencing coverage depth was at least 30X per sample.

### Epilepsy multi-gene panel testing design

A literature review yielded 111 genes, including 50 potential candidate genes for common GGE syndromes and 61 causative genes for other genetic epilepsies with absence, myoclonic, and generalized tonic-clonic seizures.

The 50 potential genes included 6 candidate Mendelian GGE genes, such as *CACNB4*, *CASR*, *GABRA1*, *GABRD*, *CLCN2*, and *EFHC1*; 9 candidate genes based on rare copy-number variants, including *CHRNA7(15q13*.*3)*, *CYFIP1(15q11*.*2)*, *NDE1(16p13*.*11)*, *GJA8(1q21*.*1)*, *CYTSB(17p11*.*2)*, *CHRM3(1q43)*, *PLCB1(20p12*.*3)*, *NRXN1(2p16*.*3)*, and *EPM2A(6q24*.*6)*; and 35 susceptibility genes from association studies (*BRD2*, *CX36*, *ME2*, *VRK2*, *ZEB2*, *SCN1A*, *PNPO*, *KCNJ10*, *CPA6*, *TAP-1*, *SCN1B*, *EFHC2*, *JRK/JH8*, *GRM4*, *CHRNA4*, *CHRNA2*, *CHRNB2*, *SCN2A*, *KCNQ3*, *HCN1*, *CHD2*, *GABRB3*, *GABRG2*, *CACNA1A*, *LGI4*, *SLC2A1/GLUT1*, *GPHN*, *CACNA1G*, *CACNA1H*, *CACNA2D2*, *GRIK1*, *KCNMA1*, *OPRM1*, *GRIN2A*, and *CHRNB3*). Detailed features of the 111 genes are described in S1 Table. The size of this region was 344.061 kbp, and the number of probes totaled 6,831, with a size of 474.708 kbp. This panel of genes includes coding exon regions as well as exon–intron boundaries of the region extensions, with 10 bases from the 3′ end and 10 bases from the 5′ end.

### Epilepsy multi-gene panel testing and data generation

Genomic DNA was extracted from peripheral blood leukocytes. The Agilent SureSelect Target Enrichment protocol from the Illumina paired-end sequencing library (v. 2.0.1, May 2010), with 1 μg input DNA, was used to generate a standard exome capture library. Sequencing was performed using the HiSeq™ 2000 platform (Illumina, San Diego, USA) at Macrogen (Seoul, Republic of Korea). The quality of the whole genome sequencing reads from all samples was assessed using FastQC, and a raw data quality control process was initiated with Trimmomatic. Alignment of the sequence reads, indexing of the reference genome hg19 from the University of California Santa Cruz (original GRCh37 from the National Center for Biotechnology Information, Feb. 2009), and variant calling with a pipeline were performed using the Genome Analysis Tool Kit best practice guidelines. Alignment was performed with the Burrows-Wheeler alignment tool (version 0.7.12) and duplicate reads were marked with Picard (version 1.130, http://broadinstitute.github.io/picard/). Local alignment, base quality recalibration, and variant calling were performed using the Genome Analysis Tool Kit (version 3.4.0), and annotation was conducted with SnpEff (version 4.1 g) at the Bioinformatics Institute of Macrogen. Low-quality variants were manually filtered. Variants with an allele depth < 10 were excluded, and multi-alleles at the same chromosomal position were removed. The alternate read ratio was calculated as alternative allele depth/total depth. Therefore, a boundary of allele ratios between 0.4 and 0.6 was established, within which single-nucleotide polymorphisms (SNPs) would be considered heterozygous for alternate reads. Allele ratios of 1 were considered homozygous for alternate reads.

### Identification of candidate or susceptibility genes or variants from sequence data

#### Identification of pathogenic variants

Disease-associated single rare variants with pathogenic effects were identified according to the American College of Medical Genetics and Genomics (ACMG) guidelines [12]. In the present study, rare variants were defined as those with minor allele frequencies (MAFs) ≤ 0.05. Rare variants were selected according to the population frequency recorded in public databases, including 1000Gp3 (1000 Genomes Project Phase 3) and ExAC (Exome Aggregation Consortium). Considering differences in allele frequencies between ethnic groups, allele frequencies were also determined via the KRGDB. To assess the variants found in patients with disease and pathogenicity, we evaluated the ClinVar and Human Gene Mutation Database (HGMD) professional version 2017. For each single rare variant, we compared differences in the frequency between the patient and control group, using Fisher's exact test. A *p* value < 0.05 was considered significant. To predict whether an amino acid substitution in a protein affects protein function, missense variants were annotated using the following protein function prediction tools: SIFT, PolyPhen-2 HDIV, PolyPhen-2 HVAR, LRT, MutationTaster, MutationAssessor, FATHMM, PROVEA, MetaSVM, and MetaLR. Four nucleotide conservation prediction tools including PhastCons, GERP, PhyloP, and SiPhy contributed to the predictions.

#### Identification of susceptibility variants using case control association analyses

Non-Mendelian genetics are commonly used to explain GGE syndromes, wherein multiple susceptibility alleles exert small effects on the occurrence of disease. To determine susceptibility alleles, case control genetic association analyses were performed. We selected all coding single-nucleotide variants (SNVs). The allele frequency of coding SNVs was compared between the disease group and 1100 control group from KRGDB. The *p* values were calculated using Fisher's exact tests for SNVs and control group. The obtained *p* values for each SNV were then summarized via Manhattan plots. The y-axis of a Manhattan plot typically represents the negative log (base 10) of the *p* values obtained for the association tests applied. The x-axis is typically organized by chromosome (chromosome 1 to 22, X). Each dot on the Manhattan plot signifies the mean for each SNV. We identified SNVs with *p* cut-off values of 0.05 (-log_10_*p* = 1.3) and corrected *p*-values using several variants.

#### Family analyses in familial cases

Genes with rare variations that segregate in association with Mendelian common GGE syndromes were identified for each familial case via family analyses. All affected family member samples underwent multi-gene panel testing. Rare variant candidates were selected using ACMG guidelines. Sanger confirmations were conducted for identified variants in families.

## Results

### Clinical description

**Fig 1:**
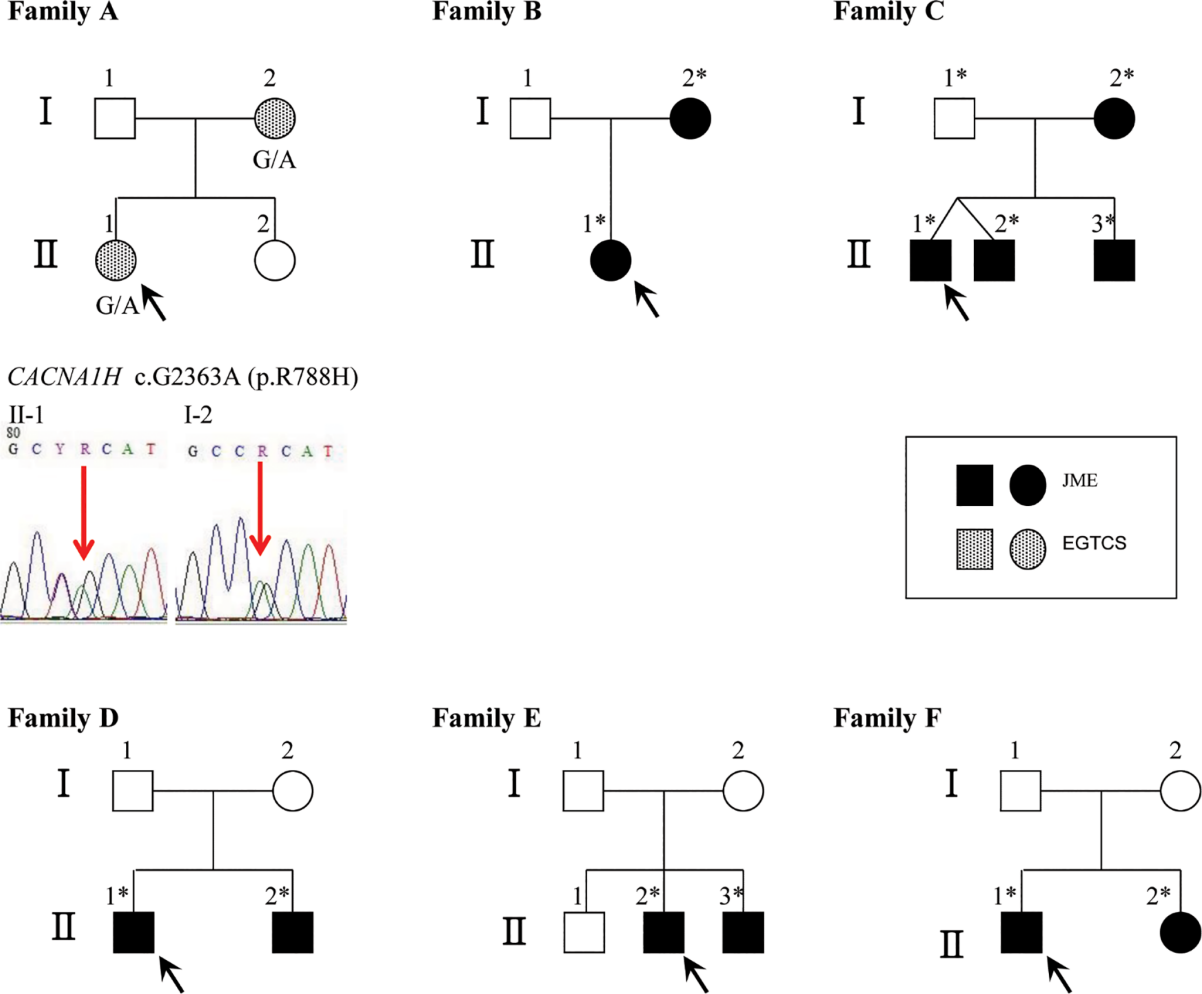
Pedigree of familial cases including 6 families and 14 patients. Black arrows indicate probands. Darkened symbols represent affected members. Stars indicate participants in the multi-gene panel testing. Vertical arrows indicate variant sites in the sequencing chromatograms. Sanger sequencing confirmed the *CACNA1H* heterozygous variant c.2363G>A, p.(Arg788His) in 2 patients (I-2 and II-1) from Family A. JME, juvenile myoclonic epilepsy; EGTCS, epilepsy with generalized tonic-clonic seizures.

A total of 57 patients with common IGE/GGE syndromes were enrolled in the present study, including 51 patients with sporadic disease and 6 with familial disease. Age at onset of seizures ranged from 5–34 years. Table 1 presents clinical data for the 57 patients. The pedigrees of the 6 familial groups are presented in Fig 1. Additionally, 8 affected family members from the 6 familial groups were included in the analyses. Thus, epilepsy multi-gene panel testing was performed for a total of 65 patients.

**Table 1:** Clinical data from 57 patients.

Trait		Results
Patients		57
Sex (M: F)		1.3:1 (32:25)
Average age at inclusion ± SD (years)		18.7 ± 2.0 (range, 14–23)
Average age at onset ± SD (years)		13.4 ± 2.7 (range, 5.2–18.4)
History of febrile seizure		7 (12.3%)
History of previous other type epilepsy		3 (5.3%)
Sporadic: Familial		51:6
Common IGE/GGEs sub-syndrome	JME	45 (79.0%)
	JAE	7 (12.3%)
	EGTCS	5 (8.8%)
EEG findings	bilateral (poly)spike and slow wave	47/57 (82.5%)
	single focal spike/sharp wave	31/57 (54.4%)
	intermittent slow activity	15/57 (26.3%)
	photoparoxysmal response	5/57 (8.8%)

M: male; F: female; IGE: idiopathic generalized epilepsy; GGE: generalized genetic epilepsy; JME: juvenile myoclonic epilepsy; JAE: juvenile absence epilepsy; EGTCS: epilepsy with generalized tonic-clonic seizures alone; EEG: electroencephalography.

### Statistical results from sequence data

**Fig 2:**
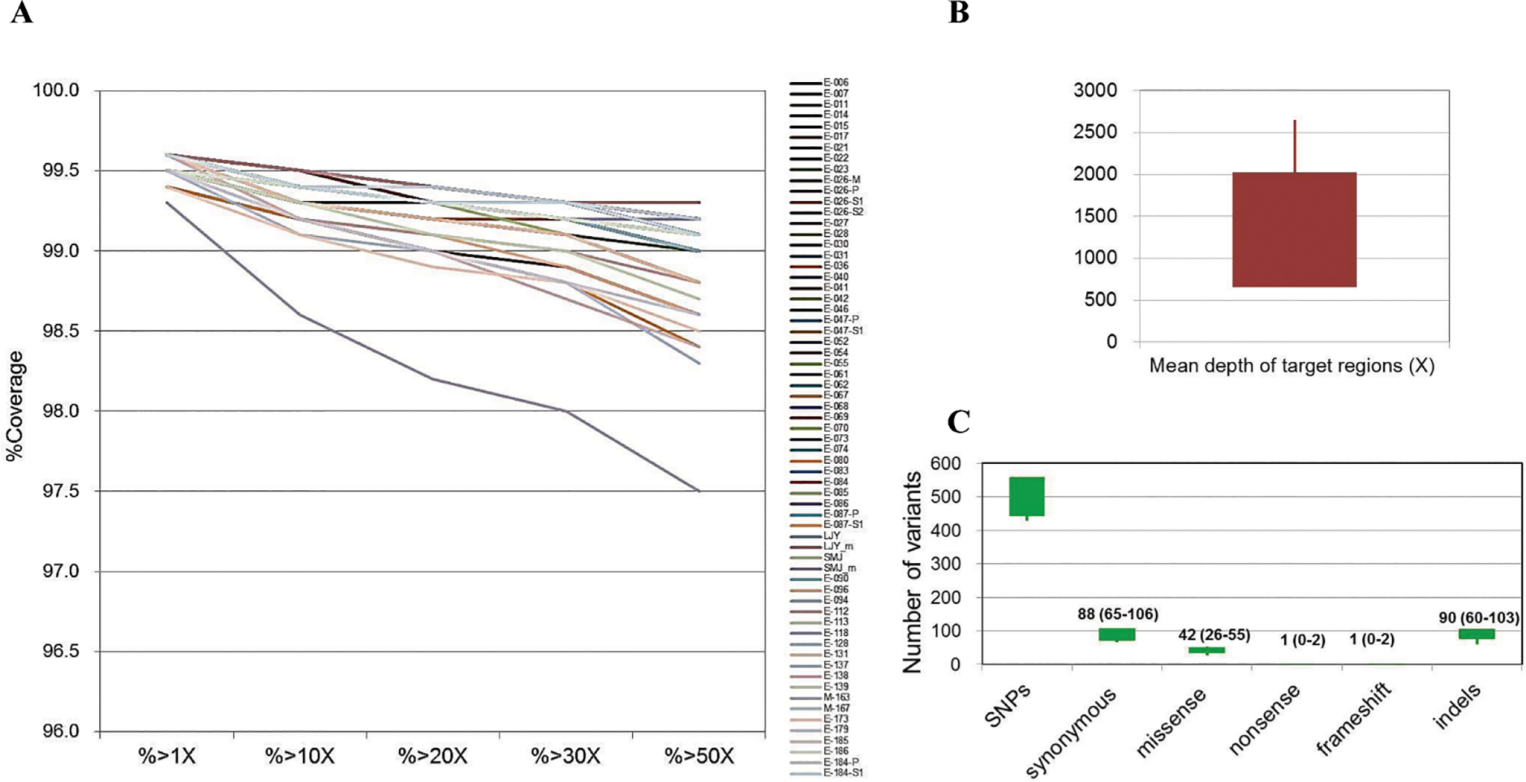
Cumulative depth distribution in target regions for each sample from 65 patients. The x-axis denotes sequencing depth, and the y-axis indicates the fraction of bases at or above a given sequencing depth. More than 97.5% of target regions were covered by more than 50 reads (A). The mean coverage depth is shown as a mean of 1342, 2 standard deviations of 682, and a range from 825 to 2649 (B). The total number of single nucleotide polymorphisms (SNPs), coding SNPs, and indels are shown (C).

An average of 13 million reads per patient were generated. Each read was approximately 101 bp long; the average total targeted region size was 340,412 bp. Approximately 99.8% of the mapped reads included the targeted regions. The mean depth was 1342 X. Table 2 and Fig 2 summarize the statistical results of the sequence data and quality controls.

**Table 2:** Summary of the statistics of multi-gene panel testing in the 65 studied patients.

Statistics	Results
Average total number of reads	13,239,759
Average read length (bp)	101
Average total yield (Mbp)	1,337
Average target regions (bp)	340,412
Average throughput depth of target regions (X)	3931,6
Average initial mappable reads	13.224.255
Average % initial mappable reads	99.8%
On-target reads	6,134,209
Average % on-target reads	54%
Average of mean depth of target regions (X)	1342.0
Average % of coverage (more than 10X)	99.4%
Average of total number of SNPs	500.6 (range, 429–554)
Average of transition/ transversion	2.4
Average Het/Hom Ratio	1.22
Average number of % found in dbSNP142	96%
Average of total number of missense variant	42.0 (range, 26–52)
Average of total number of Indels	90 (range, 60–103)

bp: base pair; Mbp: mega base pairs; SNP: single nucleotide polymorphism; Indels: small insertional-deletional variations.

### Identification of pathogenic variants

There were 3 variants that were classified pathogenic, including *GPR98* p.(Arg3227*), *CPA6* p.(Trp19Glyfs*4), and *CACNA1A* p.(Ile239Phefs*5). There were 19 variants that were classified likely pathogenic, including *CHRNB3* p.(*459Ileext*4), *GABRD* p.(Ser230Ala), *SZT2* p.(Ala1325Ser), *SZT2* p.(Leu3266Pro), *DOCK7* p.(Val1216Ala), *SCN1A* p.(Ala1440Gly), *GRM4* p.(Asn454Tyr), *GRM4* p.(Pro30Ser), *GATM* p.(Asp264Asn), *POLG* p.(Thr251Ser), *CACNA1H* p.(Gly59Cys), *CACNA1H* p.(Pro2196Thr), *CACNA1H* p.(Lys2335Arg), *CACNA1H* p.Arg788His, *NDE1* p.(Glu153Lys), *CACNA1G* p.(Ala2166Thr), *CACNA1A* p.(Glu735Ala), *LGI4* p.(Arg375Trp), and *ARX* p.(Ala283Thr). The 22 pathogenic and likely pathogenic variants were sporadically observed in 28% (16/57) of patients. Of these pathogenic and likely pathogenic variants, the most frequently identified genes were *CACNA1H* and voltage-gated Ca^2+^ channel genes including *CACNA1A*, *CACNA1G*, and *CACNA1H*, which were observed in 32% (7/22) of patients. The three pathogenic and 19 likely pathogenic variants are summarized in S2 Table. We classified 109 variants with uncertain significance and 17 likely benign variants from the 148 variants identified. Characteristics of those 109 variants with uncertain significance are summarized in S3 Table. Additionally, 96.5% (55/57) of patients had >1 variant that was classified as pathogenic, likely pathogenic, or of unknown significance. An average of 2.8 (range 0–7) variants other than likely benign variants were identified in each patient. Variant distributions by patient are summarized in Table 3.

**Table 3:** Distribution of the 131 rare variants in the 57 patients.

N	Pt ID	Phenotype	N of variants (P:LP:US)	1	2	3	4	5	6	7
1	E-006	JME	4 (0:2:2)	*LGI4*p.Arg375Tr	*SZT2* p.Leu3266Pro	*PCDH19* p.Asn1134His	*UBE3A* p.Arg279Gln			
2	E-007	JME	3 (0:0:3)	*JRK* p.Gln434Arg	*NRXN1* p.Thr1394Ser	*CHRNA4* p.Thr614Met				
3	E-011	JME	7 (0:1:6)	*ARX* p.Ala283Thr	*CACNA1H* p.His1827Tyr	*CHRNA2* p.Ser358Ile	*GPR98* p.Ser5480Asn	*KCNQ3* p.Val640Met	*SCN9A* p.Arg1893His	*SLC25A22* p.Asp51Asn
4	E-015	JME c PPR, FS	1 (0:0:1)	*SCN3A* p.Arg534Ser						
5	E-017	JME	4 (0:0:4)	*CACNA1H* p.Ala1594Ser	*CACNA1H* p.Ala1594Val	*GPR98* p.Gln4839Glu	*JRK* p.Val521Met			
6	E-021	JME, FS	3 (0:1:2)	*SCN1A* p.Ala1440Gly	*GPR98* p.Gln4839Glu	*SZT2* p.Arg1156His				
7	E-023	JME	7 (0:3:4)	*CACNA1A* p.Glu735Ala	*CACNA1H* p.Pro2196Thr	*NDE1* p.Glu153Lys	*SCN3A* p.Arg534Ser	*CHRNA4* p.Arg487Trp	*GPR98* p.Gln4839Glu	*LGI1* p.Arg407His
8	E-026-P	JME, familial	3 (0:0:3)	*CHRNA7* p.Ala153Thr	*EPM2A* p.Ala46Pro	*SCN9A* p.Met787Val				
9	E-027	JME, FS	7 (0:1:6)	*SZT2* p.Ala1325Ser	*EPM2A* p.Ala46Pro	*GPR98* p.Asn6201His	*GRIN2A* p.Val187Ile	*KCNMA1* p.Glu134Gln	*MFSD8* p.Pro69Leu	*OPRM1* p.Arg390Cys
10	E-030	JME, FS	3 (1:0:2)	*GPR98* p.Arg3227*	*EPM2A* p.Ala46Pro	*ZEB2* p.Glu668Asp				
1	E-031	JME	4 (0:0:4)	*CHD2* p.Val1346Leu	*CHRNB3* p.Val44Phe	*FOLR1* p.Arg98Trp	*SCN9A* p.Met787Val			
12	E-036	JME, FS	4 (0:0:4)	*CACNA2D* p.Lys706Glu	*CHRNA2* p.Arg382Trp	*LIAS* p.His264Tyr	*SCN8A* p.Tyr292Cys			
13	E-040	JME	5 (0:1:4)	*GABRD* p.Ser230Ala	*EFHC2* p.Arg486His	*EPM2A* p.Ala46Pro	*KCNMA1* p.Glu134Gln	*PNKP* p.Ala19Val		
14	E-041	JME	3 (0:0:3)	*GJA8* p.Gly119Glu	*LIAS* p.Ala135Thr	*SPECC1* p.Lys234Arg				
15	E-042	JME	1 (0:0:1)	*SCN9A* p.Met787Val						
16	E-047-P	JME, familial	2 (1:0:1)	*CACNA1A* p.Leu238fs	*JRK* p.Ser388Leu					
17	E-052	JME	1 (0:0:1)	*PCDH19* p.Asn1134His						
18	E-054	JME c PPR	3 (0:0:3)	*EPM2A* p.Ala46Pro	*SCN1B* p.Cys262Tyr	*SPTAN1* p.Lys2005Arg				
19	E-055	JME, FS	3 (0:0:3)	*CACNA1A* p.Arg1060Cys	*GRM4* p.Arg351His	*LGI4* p.Arg256Cys				
20	E-061	JME	0 (0:0:0)							
21	E-062	JME	3 (0:0:3)	*ALDH7A1* p.Ile21Met	*CHD2* p.Lys1491Arg	*OPRM1* p.Val386Ile				
22	E-067	JME	4 (1:1:2)	*CPA6* p.Cys18fs	*CACNA1H* p.Lys2335Arg	*CACNA1G* p.Gly674Arg	*GPR98* p.Ile4666Val			
23	E-068	JME	2 (0:1:1)	*GRM4* p.Asn454Tyr	*NDE1* p.Thr202Ala					
24	E-069	JME	3 (0:0:3)	*EPM2A* p.Ala46Pro	*PRRT2* p.Lys207Asn	*TBC1D24* p.Cys8Arg				
25	E-070	JME c PPR	2 (0:0:2)	*GPR98* p.Gly5678Ala	*PRICKLE1* p.Ala541Ser					
26	E-073	JME c PPR	1 (0:0:1)	*SCN3A* p.Arg534Ser						
27	E-074	JME	4 (0:0:4)	*CASR* p.Asn602Ser	*OPRM1* p.Arg390Cys	*PRICKLE1* p.Gly732Arg	*SCN3A* p.Lys1948Thr			
28	E-080	JME	6 (0:1:5)	*GATM* p.Asp264Asn	*CASR* p.Glu952Lys	*ME2* p.Ile478Val	*NRXN1* p.Ile1175Val	*PCDH19* p.Arg1107His	*PCDH19* p.Asn1134His	
29	E-083	JME	3 (0:0:3)	*CACNB4* p.Arg29Gln	*EFHC2* p.Ile470Val	*NRXN1* p.Val1254Ile				
30	E-084	JME	0 (0:0:0)							
31	E-085	JME	2 (0:0:2)	*CLCN2* p.Gly569Ser	*UBE3A* p.Ala201Thr					
32	E-086	JME	1 (0:0:1)	*TPP1* p.Arg350Gln						
33	E-087-P	JME, familial	1 (0:0:1)	*GPR98* p.Gly4447Asp						
34	E-090	JME c PPR	1 (0:0:1)	*SCN2A* p.Phe328Val						
35	E-093	JME	5 (0:2:3)	*CACNA1G* p.Ala2166Thr	*GRM4* p.Pro30Ser	*EPM2A* p.Ala46Pro	*KCNMA1* p.Glu134Gln	*PNKP* p.Glu337Gln		
36	E-094	JME	4 (0:0:4)	*ALDH7A1* p.His339Arg	*CACNA1G* p.Ala1099Thr	*CACNA1G* p.Arg1243Gln	*NRXN1* p.Leu204Gln			
37	E-113	JME	3 (0:0:3)	*EFHC1* p.Thr508Arg	*JRK* p.Ala362Thr	*SCN1B* p.Thr189Met				
38	E-118	JME	3 (0:0:3)	*PCDH19* p.Asn1134His	*PRICKLE1* p.Pro38Leu	*STXBP1* p.Ile427Met				
39	E-128	JME	3 (0:0:3)	*CHRNB3* p.Val44Phe	*EPM2A* p.Ala46Pro	*GPR98* p.Ser1568Asn				
40	E-137	JME	1 (0:0:1)	*CACNA1A* p.Ala1083Asp	40	E-137				
41	E-138	JME	2 (0:0:2)	*CACNA1A* p.Asn390Asp	*CHRNB2* p.Gln397Pro					
42	E-167	JME	6 (0:1:5:0)	*CACNA1H* p.Gly59Cys	*EFHC2* p.Arg132Trp	*EPM2A* p.Ala46Pro	*GABRB3* p.Leu6Pro	*ME2* p.Ile478Val	*SCN3A* p.Asp1803Asn	
43	E-173	JME	1 (0:0:1:0)	*SCN9A* p.Ser606Arg						
44	E-184-P	JME, familial	2 (0:0:2)	*ALDH7A1* p.His339Arg	*ME2* p.Ile478Val					
45	LJY	JME, familial	0 (0:0:0)							
46	E-014	JAE	4 (0:0:4)	*TBC1D24* p.Val201Met	*CACNA1A* p.Arg1060Cys	*GPR98* p.Glu777Ala	*SPECC1* p.Gly18Ser			
47	E-139	JAE	2 (0:0:2)	*GPR98* p.Ala5513Thr	*SCN1A* p.Arg1575Cys					
48	E-179	JAE	3 (0:0:3)	*CHRNA7* p.Ala153Thr	*EPM2A* p.Ala46Pro	*GPR98* p.Ile2187Val				
49	E-186	JAE	4 (0:1:3)	*CHRNB3* p.Ter459fs	*CHD2* p.Asn1625Ser	*NRXN1* p.Arg697Gln	*SPTAN1* p.Asn1034Ser			
50	E-131	JAE c CAE	3 (0:0:3)	*GRIK1* p.Arg862Gln	*PCDH19* p.Arg1107His	*PCDH19* p.Asn1134His				
51	E-163	JAE c CAE	5 (0:2:3)	*POLG* p.Thr251Ser	*DOCK7* p.Val1216Ala	*GPR98* p.Tyr4235Cys	*GPR98* p.Glu5098Ala	*LIAS* p.Ala135Thr		
52	E-185	JAE c CAE	1 (0:0:1)	*EPM2A* p.Ala46Pro						
53	SMJ	EGTCS, familial	3 (0:0:3)	*CACNA1H* p.Arg788His	*KCNQ2* p.Tyr755Cys	*ME2* p.Ile478Val				
54	E-022	EGTCS, FS	3 (0:0:3:0)	*GABRG* p.Ser8Arg	*GPR98* p.Ile1924Thr	*SCN9A* p.Met787Val				
55	E-028	EGTCS	3 (0:0:3)	*GABRD* p.Thr401Met	*GRM4* p.Pro854Thr	*TBC1D24* p.Cys8Arg				
56	E-046	EGTCS	2 (0:0:2)	*CNTNAP* p.Lys540Asn	*PNKP* p.Arg224Cys					
57	E-112	EGTCS	1 (0:0:1)	*CLCN2* p.Glu664Val						

N: number; Pt ID: Patient Identification; LP: likely pathogenic; P: pathogenic; US: uncertain significance; JME: juvenile myoclonic epilepsy; JAE: juvenile absence epilepsy; EGTCS: epilepsy with generalized tonic-clonic seizures alone; CAE: childhood absence epilepsy; PPR: photoparoxysmal response; FS: febrile seizures.

### Identification of susceptibility variants using case-control association analyses

**Fig 3:**
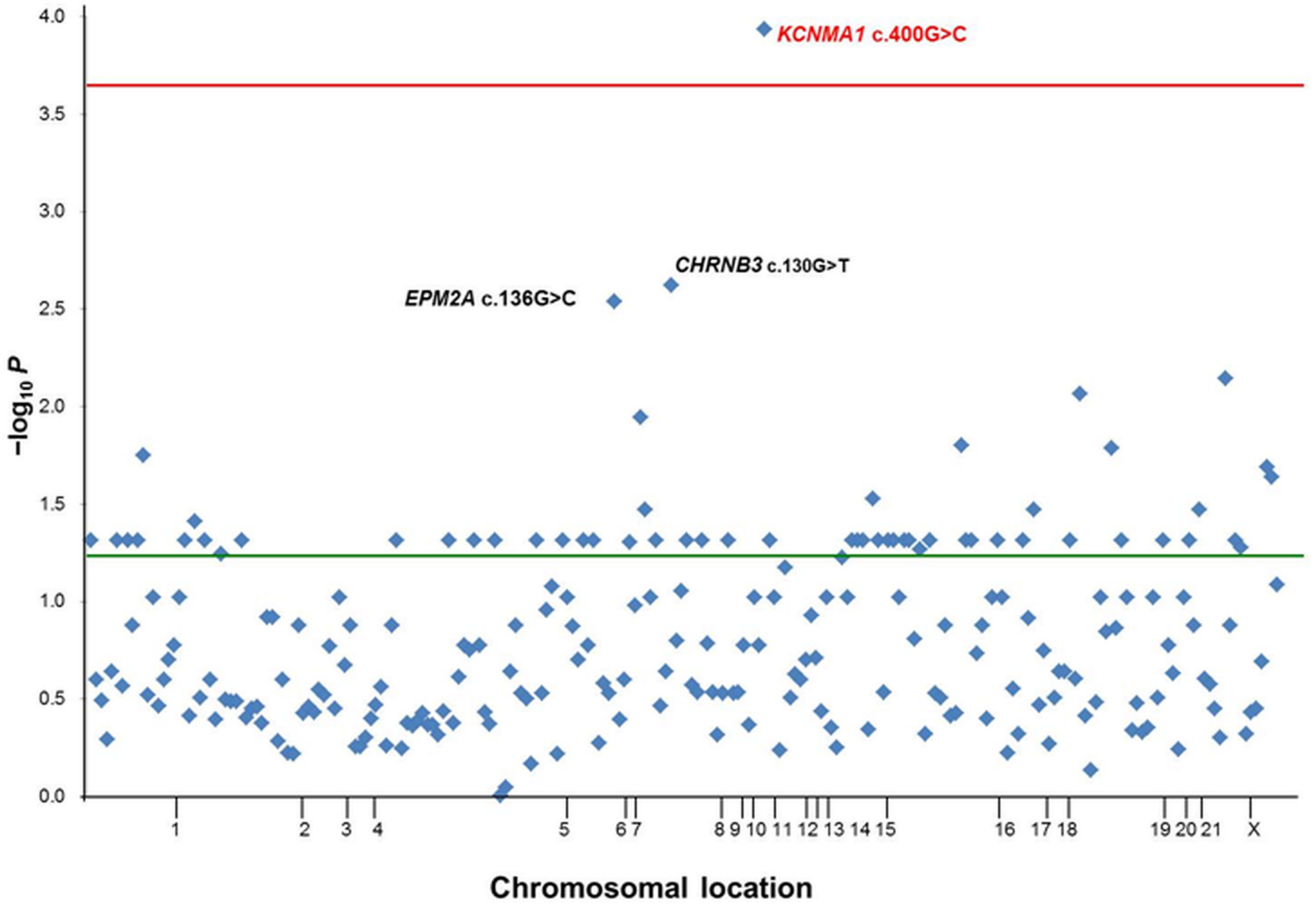
Identification of susceptibility variants using case-control analysis. Fisher's exact test *p* values calculated for 231 variants in 57 patients and 1100 participants in control group were included. Variants with statically significant *p* values ≤ 0.05 (-log_10_*p* = 1.3) and corrected *p* values of 0.0002 (-log_10_*p* = 3.7) were identified. Manhattan plots showing *p* values from the 57 patients and control groups of 1100 participants are shown. The plot shows–log_10_ *p* values (y axis) for each of the 231 single nucleotide polymorphisms against the chromosomal location (x axis). The red horizontal line indicates the significance threshold for a corrected *p* value of 0.0002, and the green horizontal line indicates a threshold for the 0.05 *p* value. SNVs = single nucleotide variations.

In total, 231 variants that were predicted to disrupt protein coding sequencing in 57 patients. The *p* values and -log_10_ (*p*) values are plotted against the chromosomal location of each gene (hg19) in Fig 3. SNVs with cut-off values of *p* ≤ 0.05 (-log_10_*p* = 1.3) were identified and adjusted to a corrected *p* value of 0.0002 (0.05/231, -log_10_*p* = 3.7). Comparisons of the 57 patients with the 1100 control participants revealed 55 variants that were significantly associated, with a *p* value of 0.05 (-log_10_*p* = 1.3). One variant, *KCNMA1* c.400G>C, was significantly associated, with a *p* value of 0.002 (-log_10_*p* = 3.7).

### Family analyses in 6 familial cases

#### Family A

An 18-year-old woman (ID: SMJ) was first assessed at 13 years of age for unprovoked GTCSs upon waking. The pedigree is illustrated in Fig 1. Her mother first experienced seizures at 25 years of age and received antiepileptic treatment with lamotrigine. Neither the proband nor her mother experienced any febrile generalized seizures. Morning myoclonic and absence seizures were not reported in either subject. The common GGE subtype diagnosis was EGTCS. Considering the autosomal dominant inheritance pattern and filtered heterozygous variants, the missense variant c.2363G>A (rs532296550) p.(Arg788His) in *CACNA1H* was strongly supported as the disease-causing variant, on the basis of the reference sequence NM_021098.2. Characteristics of the *CACNA1H* p.(Arg788His) variant are summarized in S2 Table and Table 4. The proband also had a heterozygous variant, c.2362C>T p.(Arg788Cys) in *CACNA1H*, which is known as a functional polymorphism that alters T-type calcium channels. The results of Sanger sequencing are presented in Fig 1 [13].

**Table 4:** Summary of the candidate variants in the 6 familial cases.

Family (proband)	Phenotype	Inheritance	Gene	Isoform	Nucleotide	Amino acid	Zygosity	ACMG classification	Reference
A (SMJ)	EGTCS	AD	*CACNA1H*	*NM_021098*.*2*	c.2363G>A	p.(Arg788His)	Het	Likely pathogenic	Confirmed by Sanger sequencing shown in Fig 1. No. 14 variant in S2 Table.
B (LJY)	JME	AD	No candidates						
C (E-026-P)	JME	AD	No candidates						
D (E-047-P)	JME	AR	*CACNA1A*	*NM_001127221*.*1*	c.714delT	p.(Ile239Phefs*5)	Het	Pathogenic	Sibling (E-047-S1) is negative. No. 19 variant in S2 Table.
			*JRK*	*NM_003724*.*3*	c.1163C>T	p.(Ser388Leu)	Het	Uncertain significance	Sibling (E-047-S1) had also heterozygous variant. Confirmed by Sanger sequencing. No. 57 variant in S3 Table.
E (E-087-P)	JME	AR	*GPR98*	*NM_032119*.*3*	c.13340G>A	p.(Gly4447Asp)	Het	Uncertain significance	Sibling (E-087-S1) had also heterozygous variant. Confirmed by Sanger sequencing. No. 33 variant in S3 Table.
F (E-184-P)	JME	AR	No candidates						

JME: juvenile myoclonic epilepsy; EGTCS: epilepsy with generalized tonic-clonic seizures alone; AD: autosomal dominant; AR: autosomal recessive; Het: heterozygous; US: Uncertain significance.

#### Family B

A 15-year-old girl (ID: LJY) presented with unprovoked GTCSs. She experienced unprovoked seizures since the age of 14 years that were characterized by brief episodes of myoclonic jerks, especially in the upper limbs, and GTCSs upon waking. A family history indicated epilepsy in her mother, who was diagnosed with JME. The mother was 40 years and had received treatment with an antiepileptic drug for approximately 3 years in her late teens and early twenties. The proband and her mother had no history of febrile convulsions. Given the autosomal dominant inheritance pattern and filtered heterozygous variants, no likely candidates were identified.

#### Family C

A 17-year-old boy (ID: E-026-P) had a history of recurrent myoclonic jerks, predominantly upon awakening, and GTCSs that first appeared at 8 years of age. He was diagnosed with JME. His mother, dizygotic twin brother, and younger brother had similar clinical seizures, were also diagnosed with familial JME. There was no family history of febrile seizures. The proband’s father was unaffected, and his DNA was obtained for control sequencing. Autosomal dominant inheritance from the mother was suspected; therefore, we filtered the variants. However, no candidate variants were identified.

#### Family D

A 20-year-old man (ID: E-047-P) was confirmed to have JME. He first experienced myoclonic jerks and GTCSs upon waking at 8 years of age. His younger brother also developed similar seizures (myoclonic jerks and GTCSs) at 12 years of age, was also diagnosed with JME. Both patients did not have any history of febrile seizures. Considering the autosomal recessive inheritance pattern, homozygous variants were filtered; however, no candidate variants were identified. Although it did not fit the inheritance pattern, a rare heterozygous variant *JRK* p.(Ser388Leu) was observed in both affected family members. The characteristics of the *JRK* p.(Ser388Leu) variant are described in S3 Table and Table 4. The proband also had the rare variant, *CACNA1A* p.(Ile239Phefs*5), which was suspected as possibly pathogenic; however, his sibling did not have this variant S2 Table and Table 4.

#### Family E

A 19-year-old man (ID: E-087-P) was diagnosed with JME. He first experienced myoclonic jerks and GTCSs upon waking at 17 years of age. His younger brother was also diagnosed with JME, which developed at 12 years of age. Neither patient had a history of febrile seizures. He was treated with levetiracetam and his younger brother was treated with valproate. Considering the autosomal recessive inheritance pattern, homozygous variants were filtered; however, no candidate variants were identified. Although it did not fit the inheritance pattern, the rare heterozygous variant *GPR98* p.(Gly4447Asp), which is suspected of potentially causing the disease, was observed in both affected members. Characteristics of the *GPR98* p.(Gly4447Asp) are illustrated in S3 Table and Table 4.

#### Family F

A 23-year-old man (ID: E-184-P) was diagnosed with JME. He first experienced myoclonic jerks and GTCSs upon waking at 13 years old. His younger sister had similar seizures that developed at 10 years old. Neither had a history of febrile seizures. Considering the autosomal recessive inheritance pattern, we filtered homozygous variants and did not identify any candidates. Further, heterozygous rare variants or possible single variants were not detected.

## Discussion

Based on significant advances in our knowledge regarding the genetics of epileptogenic mechanisms in the recent decades, the present study was designed to test the hypothesis that known epilepsy genes, rather than unrecognized new genes, are responsible for common GGE syndromes. We aimed to conduct a focused analysis of potential candidate genes for common GGE syndromes using multi-gene panel testing with higher coverage depth and reduced burden of data analysis compared to WES. Analyses to determine pathogenic variants indicated very high genetic heterogeneity. Three pathogenic variants and 19 likely pathogenic variants on 16 different genes were identified. The diagnostic yield in common GGE syndromes was 28% (*n* = 16/57). Of the 22 candidate variants, 73% (*n* = 16/22) on 10 genes, including *GABRD*, *SCN1A*, *GRM4*, *CPA6*, *CHRNB3*, *CACNA1H*, *NDE1*, *CACNA1G*, *CACNA1A*, and *LGI4*, were sites for 50 candidate variants for common GGE syndromes. Interestingly, 27% of variants (*n* = 6/22) on 6 genes (*SZT2*, *DOCK7*, *GPR98*, *GATM*, *POLG*, and *ARX*) belonged to 61 causative genes known to be associated with other forms of genetic epilepsy. The results reinforced the hypothesis that common GGE syndromes are polygenic disorders with extreme locus and allelic heterogeneity.

Variants in voltage-gated Ca^2+^ channel genes including *CACNA1A*, *CACNA1G*, and *CACNA1H* were also frequently observed and accounted for 32% (*n* = 7/22) of the candidate variants. Voltage-sensitive calcium channels mediate the entry of Ca^2+^ into excitable cells and are also involved in a variety of Ca^2+^-dependent processes [14, 15]. Voltage-gated Ca^2+^ channel genes are known as susceptibility factors for GAE with typical absence seizures [15]. In the present study, pathogenic variants and likely pathogenic variants on *CACNA1A*, *CACNA1G*, and *CACNA1H* were found in 4 patients with JME and familial EGTCS, as shown in Table 3. We suggested that voltage-gated Ca^2+^ channels are important to common GGE syndromes, as well as typical absence epilepsy.

*CACNA1H* variants (*n* = 4/22, 18%) were most frequently observed. The alpha-1H isoform produces T-type Ca^2+^ currents, and T-type Ca^2+^ channels belong to the low voltage-activated group. Particular characteristics of this type of channel include opening at highly negative potentials and voltage-dependent inactivation [14]. T-type channels serve pace-making functions in both central neurons and cardiac nodal cells and support calcium signaling in secretory cells and vascular smooth muscles [14]. They may also be involved in modulating neuronal firing patterns, which are crucial for information processing and cell growth processes [14]. *CACNA1H* activation produces T-type Ca^2+^ channels, and functional variations in *CACNA1H* are increased susceptibility to GGE [13, 16]. Previous studies have reported associations between common GGE syndromes and *CACNA1H*, especially GAE (CAE/JAE), primarily in the Chinese Han population [15, 17–19]. Eckle et al. [14] suggested that gain-of-function variants in *CACNA1H* directly increase seizure susceptibility by altering neuronal electrical properties, and indirectly increase seizure susceptibility by changing gene expression. Results of the present study suggest that *CACNA1H* c.2363G>A p.(Arg788His) is a pathogenic variant responsible for autosomal dominant EGTCS in family A. This missense change occurred at an amino acid residue in which a *CACNA1H* c.2362C>T (p.Arg816Cys) variant has been reported previously [13], and the p.Arg788Cys variant has been proposed as a functional polymorphism that occurs more frequently in people with IGEs than in the general population [13]. As such, the present study suggests that arginine 788 in *CACNA1H* is an important contributor to epilepsy.

Analyses to identify susceptibility variants using case-control association analyses indicated that 1 variant of *KCNMA1* c.400G>C is significantly associated when the corrected *p* value was considered. The *KCNMA1* gene underlies Ca^2+^ activated K^+^ channels, which are fundamental to smooth muscle tone and neuronal excitability. Diseases associated with *KCNMA1* include autism, cerebellar atrophy, and generalized epilepsy and paroxysmal dyskinesia [MIM 609446]. Further, *KCNMA1* c.400G>C may be the site of susceptibility variants in common GGE syndromes. However, results of the case-control association analyses are controversial, attributable to study limitations resulting from multi-gene panel testing and the relatively small sample size.

Although no meta-analysis study has been conducted, <1% of common GGEs are suspected to be heritable Mendelian monogenic diseases [7]. In the present study, 6 patients had familial disease and underwent additional family segregation analyses. Of the 6 families, only 1 (family A) had the candidate variant p.(Arg788His) on the *CACNA1H* gene. The other 5 familial cases (B–F) did not exhibit any causative gene that was consistent with the inheritance pattern via linkage analysis. Interestingly, the present study did not find any variants that were consistent with expected Mendelian JME inheritance, such as variants of *CACNB4*, *CASR*, *GABRA1*, *GABRD*, *CLCN2*, or *EFHC1*. This unexpected result in familial analysis could have several causes. The possibility of monogenic disorders with variations in penetrance and expressivity of causative genes makes it difficult to identify the pathogenic variants. Despite high coverage and depth, challenges remain in the complete coverage of targeted regions, and it is impossible to identify copy number variations (CNVs) in multi-gene panel sequencing data. For recessive disorders such as those observed for families D–F, parental testing is particularly important for analyses of pathogenic variants. Unfortunately, we did not obtain parental DNA samples from families D–F. Each proband had several rare candidate variants; however, variants were not co-segregated in each family. In particular, family D and E exhibited *JRK* p.(Ser388Leu) and *GPR98* p.(Gly4447Asp), which are rare heterozygous candidate variants and were not consistent with the expected autosomal recessive inherited pattern. Recently, several genetic studies of familial GGE showed that no individual genes were significantly associated with familial GGE; however, several known epilepsy genes and variants were significantly enriched in a group of familial GGE [16, 20]. Therefore, familial cases may result from multi-genic disorders where interactions between multiple causative and susceptibility genes or variants with modest or strong effects produce sporadic cases.

In summary, we identified candidate genetic variants causing epilepsy susceptibility in about a quarter of the patients (28%, n = 16/57) in this study. An average of 2.8 variants was identified in each of the 57 patients. The results of this study reconfirm the polygenic disorder with very high locus/allelic heterogeneity of common GGE syndromes and suggest that voltage-gated Ca^2+^ channels are possibly important contributors to common GGE syndromes. Furthermore, these data suggest that common familial GGE syndromes are polygenic disorders with low penetrance and variable expressivity. Considering the possibility of ethnic and racial differences in the distribution of epilepsy susceptibility genes and an effect of genetic background on the epilepsy phenotype, it is worth noting that this study is the first analysis of a single ethnic group of Korean patients with common GGE syndromes using multi-gene panel testing. This study extends our comprehensive understanding of common GGEs.

## Supporting information

S1 Table The 111 targeted genes in the epilepsy gene panel of this study are listed. (DOCX)

S2 Table Characteristics of the identified 3 pathogenic and 19 likely pathogenic variants according to the American College of Medical Genetics and Genomics (ACMG) classification in the 57 patients. (DOCX)

S3 Table Characteristics of the isolated 109 variants of uncertain significance according to the American College of Medical Genetics and Genomics (ACMG) classification in the 57 patients. (DOCX)
