# Supplementary material for: Multi-gene panel testing in Korean patients with common genetic generalized epilepsy syndromes
Source: PLoS One. 2018 Jun 20;13(6):e0199321. doi: 10.1371/journal.pone.0199321 (PMC6010271; doi:10.1371/journal.pone.0199321)
Supplement: S1 Table — (DOCX) [file pone.0199321.s001.docx]

**S1 Table.** The list of 111 targeted genes in the epilepsy gene panel in this study.

| **No.** | **Gene Symbol** | **Function** | **Location** | **MIM#** | **Epilepsy Phenotype** | **Reference** |
| --- | --- | --- | --- | --- | --- | --- |
| 1 | *GABRA1* | Gamma-aminobutyric acid receptor subunit alpha-1 | 5q34 | 137160 | JME, CAE, DS, WS, EIEE | [1-5] |
| 2 | *GABRB3* | Gamma-aminobutyric acid receptor subunit beta-3 | 15q12 | 137192 | CAE, LGS, EIEE, WS, AS | [5, 6] |
| 3 | *GABRG2* | Gamma-aminobutyric acid receptor subunit gamma-2 | 5q34 | 137164 | CAE, IGE, FS, GEFS+ | [7, 8] |
| 4 | *GABRD* | Gamma-aminobutyric acid receptor subunit delta | 1p36.33 | 137163 | JME, IGE, RS, FS, GEFS+ | [9, 10] |
| 5 | *CACNA1A* | Calcium channel, voltage-dependent, P/Q type, alpha 1A subunit | 19p13.2 | 601011 | IGE, LGS | [11, 12] |
| 6 | *CACNB4* | Voltage-dependent L-type calcium channel subunit beta-4 | 2q23.3 | 601949 | JME, IGE | [12, 13] |
| 7 | *SCN1B* | Sodium channel subunit beta-1 | 19q13.12 | 600235 | IGE, JME, GEFS+, FS, DS | [4] |
| 8 | *CLCN2* | Chloride channel protein 2 | 3q27.1 | 600570 | IGE, JME,CAE | [14-17] |
| 9 | *GRIN2A* | Glutamate receptor, ionotropic, N-methyl D-aspartate 2A | 16p13.2 | 138253 | BCTES, LKS, RS, PPR | [18] |
| 10 | *CHRNA7* | Cholinergic receptor, nicotinic, alpha 7 | 15q13.3 | 118511 | JME, IGE | [19-22] |
| 11 | *BRD2* | Bromodomain containing 2 | 6p21.32 | 601540 | JME, PPR | [23-27] |
| 12 | *CaSR* | Calcium-sensing receptor | 3q21.1 | 601199 | IGE, JME | [28] |
| 13 | *Cx-36* | Connexin-36 | 15q14 | 607058 | JME | [29, 30] |
| 14 | *EFHC1* | EF-hand domain (C-terminal) containing 1 | 6p12.2 | 608815 | JME, GAE | [31-36] |
| 15 | *EFHC2* | EF-hand domain (C-terminal) containing 2 | Xp11.3 | 300817 | JME | [4, 37, 38] |
| 16 | *JRK/JH8* | Jerky homologue of human on chromosome 8 | 8q24 | 600131 | JME, CAE | [4, 39, 40] |
| 17 | *ME2* | Malic enzyme 2 | 18q21.2 | 154270 | JME, IGE | [41, 42] |
| 18 | *LGI4* | Leucine-rich, glioma inactivated gene 4 | 19q13.12 | 608303 | CAE, IGE,TLE | [37, 43] |
| 19 | *GRM4* | Glutamate Receptor Type 4 | 6p21.31 | 604100 | JME, IGE | [44-46] |
| 20 | *SLC2A1/GLUT1* | Solute carrier family 2, facilitated glucose transporter member 1 | 1p34.2 | 138140 | GAE, IGE | [47, 48] |
| 21 | *GPHN* | Gephyrin | 14q23.3 | 603930 | IGE, JME | [49] |
| 22 | *TAP-1* | Transporter associated with Antigen Processing 1 | 6p21.32 | 170260 | JME | [24, 50] |
| 23 | *ZEB2* | Zinc finger E-box-binding homeobox 2 | 2q22.3 | 605802 | GAE | [51] |
| 24 | *CHRM3* | Cholinergic receptor, muscarinic 3 | 1q43 | 118494 | JME | [51] |
| 25 | *PNPO* | Pyridoxine-5'-phosphate oxidase | 17q21.32 | 603287 | GAE, EIEE, WS | [51] |
| 26 | *VRK2* | Vaccinia related kinase 2 | 2p16.1 | 602169 | GAE | [51] |
| 27 | *CYFIP1* | Cytoplasmic FMR1-interacting protein 1 | 15q11.2 | 606322 | JME | [52, 53] |
| 28 | *GJA8* | Gap junction alpha-8 protein | 1q21.2 | 600897 | IGE | [53] |
| 29 | *CYTSB/SPECC1* | Sperm antigen with calponin homology and coiled-coil domains 1 | 17p11.2 | 608793 | IGE | [53] |
| 30 | *NDE1* | Nuclear distribution protein nudE homolog 1 | 16p13.11 | 609449 | JME, TLE, BCTES | [19, 53] |
| 31 | *SCN1A* | Sodium channel, voltage-gated, type I, alpha subunit | 2q24.3 | 182389 | JME, IGE, DS, FS, GEFS+, RS, LGS | [51, 54] |
| 32 | *CACNA1G* | Calcium channel, voltage-dependent, T type, alpha 1G subunit | 17q21.33 | 604065 | IGE, JME | [55] |
| 33 | *CACNA1H* | Calcium channel, voltage-dependent, T type, alpha 1H subunit | 16p13.3 | 607904 | CAE, IGE | [56, 57] |
| 34 | *CACNA2D2* | Calcium channel, voltage-dependent, alpha2delta-2 subunit | 3p21.31 | 607082 | GAE, EIEE | [12] |
| 35 | *GRIK1* | Glutamate receptor, ionotropic, kainate 1, | [21q21.3](http://omim.org/geneMap/21/36?start=-3&limit=10&highlight=36) | 138245 | JAE | [37, 58, 59] |
| 36 | *KCNMA1* | Calcium-activated potassium channel subunit alpha-1 | 10q22.3 | 600150 | GAE | [60, 61] |
| 37 | *OPRM1* | μ-opioid receptors, MU-1 | 6q25.2 | 600018 | IGE, GAE | [62, 63] |
| 38 | *CHRNB3* | Neuronal acetylcholine receptor subunit beta-3 | 8p11.21 | 118508 | IGE, NFLE | [64] |
| 39 | *CHRNA4* | Neuronal acetylcholine receptor subunit alph-4 | 20q13.33 | 118504 | JME, NFLE | [65] |
| 40 | *CHRNA2* | Neuronal acetylcholine receptor subunit alpha-2 | 8p21.2 | 118502 | IGE, NFLE, BFIS | [66] |
| 41 | *CHRNB2* | Neuronal acetylcholine receptor subunit beta-2 | 1q21.3 | 118507 | IGE, FLE | [66] |
| 42 | *KCNT1* | Potassium channel subfamily T, member 1 | [9q34.3](http://omim.org/geneMap/9/531?start=-3&limit=10&highlight=531) | 608167 | NFLE, EIEE14, MMPSI, WS |  |
| 43 | *SCN3A* | Sodium channel, voltage-gated, type III, alpha subunit | [2q24.3](http://omim.org/geneMap/2/594?start=-3&limit=10&highlight=594) | 182391 | GEFS+ |  |
| 44 | *SCN2A* | sodium channel, voltage-gated, type II, alpha subunit | [2q24.3](http://omim.org/geneMap/2/595?start=-3&limit=10&highlight=595) | 182390 | EIEE11, BFIS, BFNS, MMPSI , FS+ |  |
| 45 | *KCNQ2* | Potassium voltage-gated channel, KQT-like subfamily, member 2 | 20q13.33 | 602235 | EIEE7, BFNS, BFIS, WS, BECTS |  |
| 46 | *KCNQ3* | Potassium voltage-gated channel, KQT-like subfamily, member 3 | [8q24.22](http://omim.org/geneMap/8/473?start=-3&limit=10&highlight=473) | 02232 | IGE, BFNS, BFIS, BECTS | [66] |
| 47 | *PRRT2* | Proline-rich transmembrane protein 2 | [16p11.2](http://omim.org/geneMap/16/271?start=-3&limit=10&highlight=271) | 614386 | BFIS, FS, LKS, BECTS |  |
| 48 | *PLCB1* | Phospholipase C, beta 1 | [20p12.3](http://omim.org/geneMap/20/74?start=-3&limit=10&highlight=74) | 607120 | JME/IGE, EIEE12, WS, MMPSI | [67] |
| 49 | *STXBP1* | Syntaxin-binding protein 1 | [9q34.11](http://omim.org/geneMap/9/431?start=-3&limit=10&highlight=431) | 602926 | EIEE4, WS, DS, RS, LSG |  |
| 50 | *GNAO1* | Guanine nucleotide-binding protein G(o) subunit alpha | 16q12.2 | 139311 | EIEE17, WS |  |
| 51 | *ST3GAL3* | ST3 beta-galactoside alpha-2,3-sialyltransferase 3 | 1p34.1 | 606494 | EIEE15, WS |  |
| 52 | *DOCK7* | Dedicator of cytokinesis 7 | 1p31.3 | 615730 | EIEE23 |  |
| 53 | *NECAP1* | Necap endocytosis-associated protein 1 | 12p13.31 | 611623 | EIEE21, RS, WS, |  |
| 54 | *SPTAN1* | Spectrin, alpha, non-erythrocytic 1 | 9q34.11 | 182810 | EIEE5, WS |  |
| 55 | *SZT2* | Seizure threshold 2 homolog | 1p34.2 | 615463 | EIEE18 |  |
| 56 | *SCN8A* | Sodium channel, voltage gated, type VIII, alpha subunit | 12q13.13 | 600702 | EIEE13, WS, LGS, RS, MMPSI |  |
| 57 | *CHD8* | Chromodomain-helicase-DNA-binding protein 8 | 14q11.2 | 610528 | seizure |  |
| 58 | *DYRK1A* | Dual specificity tyrosine-phosphorylation-regulated kinase 1A | 21q22.13 | 600855 | seizure, FS |  |
| 59 | *CDKL5* | Cyclin-dependent kinase-like 5 | Xp22.13 | 300203 | EIEE2, WS, RS, LGS, seizures |  |
| 60 | *ARX* | Aristaless-related homeobox | Xp21.3 | 300382 | EIEE1, X-linked WS |  |
| 61 | *SLC25A22* | Carrier family 25 (mitochondrial carrier, glutamate) member 22 | 11p15.5 | 609302 | EIEE3, MMPSI |  |
| 62 | *PNKP* | Polynucleotide kinase 3'-phosphatase | 19q13.33 | 605610 | EIEE10 |  |
| 63 | *ARHGEF9* | Rho guanine nucleotide exchange factor 9 | Xq11.1 | 300429 | EIEE8 |  |
| 64 | *TBC1D24* | TBC1 domain family, member 24 | 16p13.3 | 613577 | EIEE16, MMPSI |  |
| 65 | *SCN9A* | Sodium channel, voltage-gated, type IX, alpha subunit | 2q24.3 | 603415 | FS, GEFS+, DS |  |
| 66 | *HCN1* | Hyperpolarization-activated cyclic nucleotide-gated K channel 1 | 5p12 | 602780 | IGE, EIEE24, RS | [68] |
| 67 | *CHD2* | Chromodomain-helicase-DNA-binding protein 2 | 15q26.1 | 602119 | JME, PPR, MMPSI, LGS, FS | [69] |
| 68 | *ALDH7A1* | Aldehyde dehydrogenase 7 family, member A1 | 5q23.2 | 107323 | PDE |  |
| 69 | *PCDH19* | Protocadherin 19 | Xq22.1 | 300460 | EIEE9, FS, GEFS+, DS, seizures |  |
| 70 | *GPR98(ADGRV1)* | G protein-coupled receptor 98 | 5q14.3 | 602851 | FS, GEFS+ |  |
| 71 | *UBE3A* | Ubiquitin-protein ligase E3A | 15q11.2 | 601623 | AS |  |
| 72 | *MECP2* | Methyl CpG binding protein 2 | Xq28 | 300005 | RS, AS, EE |  |
| 73 | *FOXG1* | Forkhead box protein G1 | 14q12 | 164874 | RS, WS, LGS |  |
| 74 | *TCF4* | Transcription factor 4 | 18q21.2 | 602272 | AS, RS |  |
| 75 | *NRXN1* | Neurexin-1-alpha | 2p16.3 | 600565 | IGE/JME, seizure, epilepsy | [67] |
| 76 | *CNTNAP2* | Contactin-associated protein-like 2 | 7q35-q36 | 604569 | epilepsy |  |
| 77 | *KCNJ10* | ATP-sensitive inward rectifier potassium channel 10 | 1q23.2 | 602208 | IGE/JME, RS, epilepsy | [70-72] |
| 78 | *ST3GAL5* | ST3 beta-galactoside alpha-2,3-sialyltransferase 5 | [2p11.2](http://omim.org/geneMap/2/356?start=-3&limit=10&highlight=356) | 604402 | epilepsy |  |
| 79 | *OPHN1* | Oligophrenin 1 | Xq12 | 300127 | epilepsy |  |
| 80 | *PHF6* | PHD finger protein 6 | Xq26.2 | 300414 | epilepsy |  |
| 81 | *SYN1* | Synapsin I | Xp11.23 | 313440 | epilepsy |  |
| 82 | *GRIN2B* | Glutamate receptor, ionotropic, N-methyl D-aspartate 2B | 12p13.1 | 138252 | EIEE27, WS, RS, LGS |  |
| 83 | *CSTB* | Cystatin-B | 21q22.3 | 601145 | PME1A |  |
| 84 | *EPM2A* | Epilepsy, progressive myoclonic 2A (Lafora) | 6q24.3 | 607566 | IGE/JME, PME2A | [67] |
| 85 | *EPM2B* | Epilepsy, progressive myoclonic 2B (Lafora), NHLRC1 | 6p22.3 | 608072 | PME2B |  |
| 86 | *SCARB2/LIMP2* | Lysosome membrane protein 2 | 4q21.1 | 602257 | PME4 |  |
| 87 | *GOSR2* | Golgi SNAP receptor complex member 2 | 17q21.32 | 604027 | PME6 |  |
| 88 | *KCTD7* | Potassium channel tetramerisation domain containing 7 | 7q11.21 | 611725 | PME3 |  |
| 89 | *PRICKLE1* | Prickle homolog 1 | 12q12 | 608500 | PME1B |  |
| 90 | *NEU1* | Sialidase 1 | 6p21.33 | 608272 | Sialidosis |  |
| 91 | *PRICKLE2* | Prickle homolog 2 | 3p14.1 | 608501 | PME5 |  |
| 92 | *CLN6* | Ceroid-lipofuscinosis neuronal protein 6 | 15q23 | 606725 | PME |  |
| 93 | *CLN3* | Ceroid-lipofuscinosis neuronal protein 3 | 16p11.2 | 607042 | myoclonus epilepsy |  |
| 94 | *CLN5* | Ceroid-lipofuscinosis neuronal protein 5 | 13q22.3 | 608102 | myoclonus epilepsy |  |
| 95 | *PPT1* | Palmitoyl-protein thioesterase 1 | 1p34.2 | 600722 | myoclonus epilepsy |  |
| 96 | *TPP1* | Tripeptidyl-peptidase 1 | 11p15.4 | 607998 | myoclonus epilepsy |  |
| 97 | *MFSD8* | Major facilitator superfamily domain containing 8 | 4q28.2 | 611124 | myoclonus epilepsy |  |
| 98 | *DNAJC5* | DnaJ homolog subfamily C member | 20q13.33 | 611203 | myoclonus epilepsy |  |
| 99 | *CTSD* | Cathepsin D | 11p15.5 | 116840 | myoclonus epilepsy |  |
| 100 | *SRPX2* | Sushi repeat-containing protein, X-linked 2 | Xq22.1 | 300642 | BCTES |  |
| 101 | *CPA6* | Carboxypeptidase A6 | 8q13.2 | 609562 | JME, TLE, FS | [73] |
| 102 | *LGI1* | Leucine-rich, glioma inactivated 1 | 10q23.33 | 604619 | TLE, epilepsy |  |
| 103 | *ABAT* | 4-Aminobutyrate aminotransferase | 16p13.2 | 137150 | epilepsy |  |
| 104 | *ADSL* | Adenylosuccinate lyase | [22q13.1](https://omim.org/geneMap/22/268?start=-3&limit=10&highlight=268) | 608222 | epilepsy, WS |  |
| 105 | *FOLR1* | Folate receptor alpha | 11q13.4 | 136430 | epilepsy |  |
| 106 | *GAMT* | Guanidinoacetate N-methyltransferase | [19p13.3](https://omim.org/geneMap/19/48?start=-3&limit=10&highlight=48) | 601240 | epilepsy |  |
| 107 | *GATM* | Glycine amidinotransferase | [15q21.1](http://www.omim.org/geneMap/15/163?start=-3&limit=10&highlight=163) | 602360 | epilepsy |  |
| 108 | *LIAS* | Lipoic acid synthetase | 4p14 | 607031 | epilepsy |  |
| 109 | *NDUFA1* | NADH dehydrogenase 1 alpha subcomplex subunit 1 | Xq24 | 300078 | epilepsy |  |
| 110 | *POLG* | DNA polymerase subunit gamma | 15q26.1 | 174763 | myoclonus epilepsy |  |
| 111 | *SLC19A3* | Solute carrier family 19, member 3 | 2q36.3 | 606152 | EIEE, epilepsy |  |

N: number; JME: juvenile myoclonic epilepsy; CAE: childhood absence epilepsy; DS: Dravet syndrome; WS: West syndrome; IGE: idiopathic generalized epilepsy; EIEE: early infantile epileptic encephalopathy; LGS: Lennox–Gastaut syndrome; AS: Angelman syndrome; FS: febrile seizures; GEFS+: generalized epilepsy with febrile seizures plus; RS: Rett syndrome; BCTES: benign rolandic epilepsy with centro-temporal spikes; LKS: Landau-Kleffner syndrome; PPR: photoparoxysmal response; TLE: temporal lobe epilepsy; GAE: generalized absence epilepsy; NFLE: nocturnal frontal lobe epilepsy; BFIS: benign familial infantile seizures; MMPSI: malignant migrating partial seizures of infancy; BFNS: benign familial neonatal seizures; PDE: pyridoxine-dependent epilepsy; PME: progressive myoclonus epilepsy.

**References**

1. Crunelli V, Leresche N. Childhood absence epilepsy: genes, channels, neurons and networks. Nature reviews Neuroscience. 2002;3(5):371-82. Epub 2002/05/04. doi: 10.1038/nrn811. PubMed PMID: 11988776.

2. Arain FM, Boyd KL, Gallagher MJ. Decreased viability and absence-like epilepsy in mice lacking or deficient in the GABAA receptor alpha1 subunit. Epilepsia. 2012;53(8):e161-5. Epub 2012/07/21. doi: 10.1111/j.1528-1167.2012.03596.x. PubMed PMID: 22812724; PubMed Central PMCID: PMCPMC3418418.

3. Cossette P, Liu L, Brisebois K, Dong H, Lortie A, Vanasse M, et al. Mutation of GABRA1 in an autosomal dominant form of juvenile myoclonic epilepsy. Nature genetics. 2002;31(2):184-9. Epub 2002/05/07. doi: 10.1038/ng885. PubMed PMID: 11992121.

4. Delgado-Escueta AV, Koeleman BP, Bailey JN, Medina MT, Duron RM. The quest for juvenile myoclonic epilepsy genes. Epilepsy & behavior : E&B. 2013;28 Suppl 1:S52-7. Epub 2013/06/14. doi: 10.1016/j.yebeh.2012.06.033. PubMed PMID: 23756480.

5. Macdonald RL, Kang JQ, Gallagher MJ. GABAA Receptor Subunit Mutations and Genetic Epilepsies. In: Noebels JL, Avoli M, Rogawski MA, Olsen RW, Delgado-Escueta AV, editors. Jasper's Basic Mechanisms of the Epilepsies. Bethesda MD: Michael A Rogawski, Antonio V Delgado-Escueta, Jeffrey L Noebels, Massimo Avoli and Richard W Olsen.; 2012.

6. Feucht M, Fuchs K, Pichlbauer E, Hornik K, Scharfetter J, Goessler R, et al. Possible association between childhood absence epilepsy and the gene encoding GABRB3. Biological psychiatry. 1999;46(7):997-1002. Epub 1999/10/06. PubMed PMID: 10509183.

7. Baulac S, Huberfeld G, Gourfinkel-An I, Mitropoulou G, Beranger A, Prud'homme JF, et al. First genetic evidence of GABA(A) receptor dysfunction in epilepsy: a mutation in the gamma2-subunit gene. Nature genetics. 2001;28(1):46-8. Epub 2001/04/28. doi: 10.1038/88254. PubMed PMID: 11326274.

8. Lachance-Touchette P, Brown P, Meloche C, Kinirons P, Lapointe L, Lacasse H, et al. Novel alpha1 and gamma2 GABAA receptor subunit mutations in families with idiopathic generalized epilepsy. The European journal of neuroscience. 2011;34(2):237-49. Epub 2011/07/01. doi: 10.1111/j.1460-9568.2011.07767.x. PubMed PMID: 21714819.

9. Dibbens LM, Feng HJ, Richards MC, Harkin LA, Hodgson BL, Scott D, et al. GABRD encoding a protein for extra- or peri-synaptic GABAA receptors is a susceptibility locus for generalized epilepsies. Human molecular genetics. 2004;13(13):1315-9. Epub 2004/04/30. doi: 10.1093/hmg/ddh146. PubMed PMID: 15115768.

10. Feng HJ, Kang JQ, Song L, Dibbens L, Mulley J, Macdonald RL. Delta subunit susceptibility variants E177A and R220H associated with complex epilepsy alter channel gating and surface expression of alpha4beta2delta GABAA receptors. The Journal of neuroscience : the official journal of the Society for Neuroscience. 2006;26(5):1499-506. Epub 2006/02/03. doi: 10.1523/jneurosci.2913-05.2006. PubMed PMID: 16452673.

11. Jouvenceau A, Eunson LH, Spauschus A, Ramesh V, Zuberi SM, Kullmann DM, et al. Human epilepsy associated with dysfunction of the brain P/Q-type calcium channel. Lancet. 2001;358(9284):801-7. Epub 2001/09/21. doi: 10.1016/s0140-6736(01)05971-2. PubMed PMID: 11564488.

12. Noebels JL. The Voltage-Gated Calcium Channel and Absence Epilepsy. In: Noebels JL, Avoli M, Rogawski MA, Olsen RW, Delgado-Escueta AV, editors. Jasper's Basic Mechanisms of the Epilepsies. Bethesda MD: Michael A Rogawski, Antonio V Delgado-Escueta, Jeffrey L Noebels, Massimo Avoli and Richard W Olsen.; 2012.

13. Escayg A, De Waard M, Lee DD, Bichet D, Wolf P, Mayer T, et al. Coding and noncoding variation of the human calcium-channel beta4-subunit gene CACNB4 in patients with idiopathic generalized epilepsy and episodic ataxia. American journal of human genetics. 2000;66(5):1531-9. Epub 2000/04/14. doi: 10.1086/302909. PubMed PMID: 10762541; PubMed Central PMCID: PMCPMC1378014.

14. Saint-Martin C, Gauvain G, Teodorescu G, Gourfinkel-An I, Fedirko E, Weber YG, et al. Two novel CLCN2 mutations accelerating chloride channel deactivation are associated with idiopathic generalized epilepsy. Human mutation. 2009;30(3):397-405. Epub 2009/02/05. doi: 10.1002/humu.20876. PubMed PMID: 19191339.

15. Stogmann E, Lichtner P, Baumgartner C, Schmied M, Hotzy C, Asmus F, et al. Mutations in the CLCN2 gene are a rare cause of idiopathic generalized epilepsy syndromes. Neurogenetics. 2006;7(4):265-8. Epub 2006/08/26. doi: 10.1007/s10048-006-0057-x. PubMed PMID: 16932951.

16. Everett K, Chioza B, Aicardi J, Aschauer H, Brouwer O, Callenbach P, et al. Linkage and mutational analysis of CLCN2 in childhood absence epilepsy. Epilepsy research. 2007;75(2-3):145-53. Epub 2007/06/21. doi: 10.1016/j.eplepsyres.2007.05.004. PubMed PMID: 17580110.

17. Heils A. CLCN2 and idiopathic generalized epilepsy. Advances in neurology. 2005;95:265-71. Epub 2004/10/29. PubMed PMID: 15508929.

18. von Spiczak S, Finsterwalder K, Muhle H, Franke A, Schilhabel M, Stephani U, et al. Comprehensive analysis of candidate genes for photosensitivity using a complementary bioinformatic and experimental approach. Epilepsia. 2011;52(10):e143-7. Epub 2011/09/03. doi: 10.1111/j.1528-1167.2011.03197.x. PubMed PMID: 21883175.

19. Jahn JA, von Spiczak S, Muhle H, Obermeier T, Franke A, Mefford HC, et al. Iterative phenotyping of 15q11.2, 15q13.3 and 16p13.11 microdeletion carriers in pediatric epilepsies. Epilepsy research. 2014;108(1):109-16. Epub 2013/11/20. doi: 10.1016/j.eplepsyres.2013.10.001. PubMed PMID: 24246141.

20. Kirov A, Dimova P, Todorova A, Mefford H, Todorov T, Saraylieva G, et al. 15q13.3 microdeletions in a prospectively recruited cohort of patients with idiopathic generalized epilepsy in Bulgaria. Epilepsy research. 2013;104(3):241-5. Epub 2013/01/29. doi: 10.1016/j.eplepsyres.2012.10.013. PubMed PMID: 23352738.

21. Helbig I, Mefford HC, Sharp AJ, Guipponi M, Fichera M, Franke A, et al. 15q13.3 microdeletions increase risk of idiopathic generalized epilepsy. Nature genetics. 2009;41(2):160-2. Epub 2009/01/13. doi: 10.1038/ng.292. PubMed PMID: 19136953; PubMed Central PMCID: PMCPMC3026630.

22. Helbig I, Hartmann C, Mefford HC. The unexpected role of copy number variations in juvenile myoclonic epilepsy. Epilepsy & behavior : E&B. 2013;28 Suppl 1:S66-8. Epub 2013/06/14. doi: 10.1016/j.yebeh.2012.07.005. PubMed PMID: 23756484.

23. de Kovel CG, Pinto D, de Haan GJ, Kasteleijn-Nolst Trenite DG, Lindhout D, Koeleman BP. Association analysis of BRD2 (RING3) and epilepsy in a Dutch population. Epilepsia. 2007;48(11):2191-2. Epub 2007/11/15. doi: 10.1111/j.1528-1167.2007.01306.x. PubMed PMID: 17999746.

24. Layouni S, Buresi C, Thomas P, Malafosse A, Dogui M. BRD2 and TAP-1 genes and juvenile myoclonic epilepsy. Neurological sciences : official journal of the Italian Neurological Society and of the Italian Society of Clinical Neurophysiology. 2010;31(1):53-6. Epub 2009/12/03. doi: 10.1007/s10072-009-0190-z. PubMed PMID: 19953286.

25. Cavalleri GL, Walley NM, Soranzo N, Mulley J, Doherty CP, Kapoor A, et al. A multicenter study of BRD2 as a risk factor for juvenile myoclonic epilepsy. Epilepsia. 2007;48(4):706-12. Epub 2007/04/18. doi: 10.1111/j.1528-1167.2007.00977.x. PubMed PMID: 17437413.

26. Pal DK, Evgrafov OV, Tabares P, Zhang F, Durner M, Greenberg DA. BRD2 (RING3) is a probable major susceptibility gene for common juvenile myoclonic epilepsy. American journal of human genetics. 2003;73(2):261-70. Epub 2003/06/28. doi: 10.1086/377006. PubMed PMID: 12830434; PubMed Central PMCID: PMCPMC1180366.

27. Lorenz S, Taylor KP, Gehrmann A, Becker T, Muhle H, Gresch M, et al. Association of BRD2 polymorphisms with photoparoxysmal response. Neuroscience letters. 2006;400(1-2):135-9. Epub 2006/03/07. doi: 10.1016/j.neulet.2006.02.026. PubMed PMID: 16516380.

28. Kapoor A, Satishchandra P, Ratnapriya R, Reddy R, Kadandale J, Shankar SK, et al. An idiopathic epilepsy syndrome linked to 3q13.3-q21 and missense mutations in the extracellular calcium sensing receptor gene. Annals of neurology. 2008;64(2):158-67. Epub 2008/08/30. doi: 10.1002/ana.21428. PubMed PMID: 18756473.

29. Mas C, Taske N, Deutsch S, Guipponi M, Thomas P, Covanis A, et al. Association of the connexin36 gene with juvenile myoclonic epilepsy. Journal of medical genetics. 2004;41(7):e93. Epub 2004/07/06. PubMed PMID: 15235036; PubMed Central PMCID: PMCPMC1735851.

30. Hempelmann A, Heils A, Sander T. Confirmatory evidence for an association of the connexin-36 gene with juvenile myoclonic epilepsy. Epilepsy research. 2006;71(2-3):223-8. Epub 2006/08/01. doi: 10.1016/j.eplepsyres.2006.06.021. PubMed PMID: 16876983.

31. Suzuki T, Delgado-Escueta AV, Aguan K, Alonso ME, Shi J, Hara Y, et al. Mutations in EFHC1 cause juvenile myoclonic epilepsy. Nature genetics. 2004;36(8):842-9. Epub 2004/07/20. doi: 10.1038/ng1393. PubMed PMID: 15258581.

32. de Nijs L, Wolkoff N, Coumans B, Delgado-Escueta AV, Grisar T, Lakaye B. Mutations of EFHC1, linked to juvenile myoclonic epilepsy, disrupt radial and tangential migrations during brain development. Human molecular genetics. 2012;21(23):5106-17. Epub 2012/08/29. doi: 10.1093/hmg/dds356. PubMed PMID: 22926142; PubMed Central PMCID: PMCPMC3490517.

33. de Nijs L, Wolkoff N, Grisar T, Lakaye B. Juvenile myoclonic epilepsy as a possible neurodevelopmental disease: role of EFHC1 or Myoclonin1. Epilepsy & behavior : E&B. 2013;28 Suppl 1:S58-60. Epub 2013/06/14. doi: 10.1016/j.yebeh.2012.06.034. PubMed PMID: 23756481.

34. Grisar T, Lakaye B, de Nijs L, LoTurco J, Daga A, Delgado-Escueta AV. Myoclonin1/EFHC1 in cell division, neuroblast migration, synapse/dendrite formation in juvenile myoclonic epilepsy. In: Noebels JL, Avoli M, Rogawski MA, Olsen RW, Delgado-Escueta AV, editors. Jasper's Basic Mechanisms of the Epilepsies. Bethesda MD: Michael A Rogawski, Antonio V Delgado-Escueta, Jeffrey L Noebels, Massimo Avoli and Richard W Olsen.; 2012.

35. Jara-Prado A, Martinez-Juarez IE, Ochoa A, Gonzalez VM, Fernandez-Gonzalez-Aragon Mdel C, Lopez-Ruiz M, et al. Novel Myoclonin1/EFHC1 mutations in Mexican patients with juvenile myoclonic epilepsy. Seizure : the journal of the British Epilepsy Association. 2012;21(7):550-4. Epub 2012/06/26. doi: 10.1016/j.seizure.2012.05.016. PubMed PMID: 22727576.

36. von Podewils F, Kowoll V, Schroeder W, Geithner J, Wang ZI, Gaida B, et al. Predictive value of EFHC1 variants for the long-term seizure outcome in juvenile myoclonic epilepsy. Epilepsy & behavior : E&B. 2015;44:61-6. Epub 2015/01/28. doi: 10.1016/j.yebeh.2014.12.016. PubMed PMID: 25625532.

37. Lucarini N, Verrotti A, Napolioni V, Bosco G, Curatolo P. Genetic polymorphisms and idiopathic generalized epilepsies. Pediatric neurology. 2007;37(3):157-64. Epub 2007/09/04. doi: 10.1016/j.pediatrneurol.2007.06.001. PubMed PMID: 17765802.

38. Gu W, Sander T, Heils A, Lenzen KP, Steinlein OK. A new EF-hand containing gene EFHC2 on Xp11.4: tentative evidence for association with juvenile myoclonic epilepsy. Epilepsy research. 2005;66(1-3):91-8. Epub 2005/08/23. doi: 10.1016/j.eplepsyres.2005.07.003. PubMed PMID: 16112844.

39. Morita R, Miyazaki E, Shah PU, Castroviejo IP, Delgado-Escueta AV, Yamakawa K. Exclusion of the JRK/JH8 gene as a candidate for human childhood absence epilepsy mapped on 8q24. Epilepsy research. 1999;37(2):151-8. Epub 1999/10/08. PubMed PMID: 10510981.

40. Moore T, Hecquet S, McLellann A, Ville D, Grid D, Picard F, et al. Polymorphism analysis of JRK/JH8, the human homologue of mouse jerky, and description of a rare mutation in a case of CAE evolving to JME. Epilepsy research. 2001;46(2):157-67. Epub 2001/07/21. PubMed PMID: 11463517.

41. Greenberg DA, Cayanis E, Strug L, Marathe S, Durner M, Pal DK, et al. Malic enzyme 2 may underlie susceptibility to adolescent-onset idiopathic generalized epilepsy. American journal of human genetics. 2005;76(1):139-46. Epub 2004/11/09. doi: 10.1086/426735. PubMed PMID: 15532013; PubMed Central PMCID: PMCPMC1196416.

42. Lenzen KP, Heils A, Lorenz S, Hempelmann A, Sander T. Association analysis of malic enzyme 2 gene polymorphisms with idiopathic generalized epilepsy. Epilepsia. 2005;46(10):1637-41. Epub 2005/09/30. doi: 10.1111/j.1528-1167.2005.00270.x. PubMed PMID: 16190936.

43. Bisulli F, Naldi I, Baldassari S, Magini P, Licchetta L, Castegnaro G, et al. Autosomal dominant partial epilepsy with auditory features: a new locus on chromosome 19q13.11-q13.31. Epilepsia. 2014;55(6):841-8. Epub 2014/03/04. doi: 10.1111/epi.12560. PubMed PMID: 24579982.

44. Muhle H, von Spiczak S, Gaus V, Kara S, Helbig I, Hampe J, et al. Role of GRM4 in idiopathic generalized epilepsies analysed by genetic association and sequence analysis. Epilepsy research. 2010;89(2-3):319-26. Epub 2010/03/27. doi: 10.1016/j.eplepsyres.2010.02.004. PubMed PMID: 20338729.

45. Izzi C, Barbon A, Toliat MR, Heils A, Becker C, Nurnberg P, et al. Candidate gene analysis of the human metabotropic glutamate receptor type 4 (GRM4) in patients with juvenile myoclonic epilepsy. American journal of medical genetics Part B, Neuropsychiatric genetics : the official publication of the International Society of Psychiatric Genetics. 2003;123B(1):59-63. Epub 2003/10/29. doi: 10.1002/ajmg.b.20024. PubMed PMID: 14582146.

46. Parihar R, Mishra R, Singh SK, Jayalakshmi S, Mehndiratta MM, Ganesh S. Association of the GRM4 gene variants with juvenile myoclonic epilepsy in an Indian population. Journal of genetics. 2014;93(1):193-7. Epub 2014/05/21. PubMed PMID: 24840839.

47. Striano P, Weber YG, Toliat MR, Schubert J, Leu C, Chaimana R, et al. GLUT1 mutations are a rare cause of familial idiopathic generalized epilepsy. Neurology. 2012;78(8):557-62. Epub 2012/01/28. doi: 10.1212/WNL.0b013e318247ff54. PubMed PMID: 22282645.

48. Lebon S, Suarez P, Alija S, Korff CM, Fluss J, Mercati D, et al. When should clinicians search for GLUT1 deficiency syndrome in childhood generalized epilepsies? European journal of paediatric neurology : EJPN : official journal of the European Paediatric Neurology Society. 2015;19(2):170-5. Epub 2014/12/24. doi: 10.1016/j.ejpn.2014.11.009. PubMed PMID: 25532859.

49. Dejanovic B, Lal D, Catarino CB, Arjune S, Belaidi AA, Trucks H, et al. Exonic microdeletions of the gephyrin gene impair GABAergic synaptic inhibition in patients with idiopathic generalized epilepsy. Neurobiology of disease. 2014;67:88-96. Epub 2014/02/25. doi: 10.1016/j.nbd.2014.02.001. PubMed PMID: 24561070.

50. Layouni S, Chouchane L, Malafosse A, Dogui M. Dimorphism of TAP-1 gene in Caucasian with juvenile myoclonic epilepsy and in Tunisian with idiopathic generalized epilepsies. International journal of immunogenetics. 2010;37(2):117-23. Epub 2010/02/10. doi: 10.1111/j.1744-313X.2010.00900.x. PubMed PMID: 20141545.

51. Steffens M, Leu C, Ruppert AK, Zara F, Striano P, Robbiano A, et al. Genome-wide association analysis of genetic generalized epilepsies implicates susceptibility loci at 1q43, 2p16.1, 2q22.3 and 17q21.32. Human molecular genetics. 2012;21(24):5359-72. Epub 2012/09/06. doi: 10.1093/hmg/dds373. PubMed PMID: 22949513.

52. de Kovel CG, Trucks H, Helbig I, Mefford HC, Baker C, Leu C, et al. Recurrent microdeletions at 15q11.2 and 16p13.11 predispose to idiopathic generalized epilepsies. Brain : a journal of neurology. 2010;133(Pt 1):23-32. Epub 2009/10/22. doi: 10.1093/brain/awp262. PubMed PMID: 19843651; PubMed Central PMCID: PMCPMC2801323.

53. Mefford HC, Muhle H, Ostertag P, von Spiczak S, Buysse K, Baker C, et al. Genome-wide copy number variation in epilepsy: novel susceptibility loci in idiopathic generalized and focal epilepsies. PLoS genetics. 2010;6(5):e1000962. Epub 2010/05/27. doi: 10.1371/journal.pgen.1000962. PubMed PMID: 20502679; PubMed Central PMCID: PMCPMC2873910.

54. Jingami N, Matsumoto R, Ito H, Ishii A, Ihara Y, Hirose S, et al. A novel SCN1A mutation in a cytoplasmic loop in intractable juvenile myoclonic epilepsy without febrile seizures. Epileptic disorders : international epilepsy journal with videotape. 2014;16(2):227-31. Epub 2014/05/21. doi: 10.1684/epd.2014.0657. PubMed PMID: 24842605.

55. Singh B, Monteil A, Bidaud I, Sugimoto Y, Suzuki T, Hamano S, et al. Mutational analysis of CACNA1G in idiopathic generalized epilepsy. Mutation in brief #962. Online. Human mutation. 2007;28(5):524-5. Epub 2007/04/03. doi: 10.1002/humu.9491. PubMed PMID: 17397049.

56. Heron SE, Khosravani H, Varela D, Bladen C, Williams TC, Newman MR, et al. Extended spectrum of idiopathic generalized epilepsies associated with CACNA1H functional variants. Annals of neurology. 2007;62(6):560-8. Epub 2007/08/19. doi: 10.1002/ana.21169. PubMed PMID: 17696120.

57. Heron SE, Phillips HA, Mulley JC, Mazarib A, Neufeld MY, Berkovic SF, et al. Genetic variation of CACNA1H in idiopathic generalized epilepsy. Annals of neurology. 2004;55(4):595-6. Epub 2004/03/30. doi: 10.1002/ana.20028. PubMed PMID: 15048902.

58. Sander T, Hildmann T, Kretz R, Furst R, Sailer U, Bauer G, et al. Allelic association of juvenile absence epilepsy with a GluR5 kainate receptor gene (GRIK1) polymorphism. American journal of medical genetics. 1997;74(4):416-21. Epub 1997/07/25. PubMed PMID: 9259378.

59. Izzi C, Barbon A, Kretz R, Sander T, Barlati S. Sequencing of the GRIK1 gene in patients with juvenile absence epilepsy does not reveal mutations affecting receptor structure. American journal of medical genetics. 2002;114(3):354-9. Epub 2002/03/29. PubMed PMID: 11920863.

60. Guerrini R. Idiopathic epilepsy and paroxysmal dyskinesia. Epilepsia. 2001;42 Suppl 3:36-41. Epub 2001/08/25. PubMed PMID: 11520321.

61. Du W, Bautista JF, Yang H, Diez-Sampedro A, You SA, Wang L, et al. Calcium-sensitive potassium channelopathy in human epilepsy and paroxysmal movement disorder. Nature genetics. 2005;37(7):733-8. Epub 2005/06/07. doi: 10.1038/ng1585. PubMed PMID: 15937479.

62. Sander T, Berlin W, Gscheidel N, Wendel B, Janz D, Hoehe MR. Genetic variation of the human mu-opioid receptor and susceptibility to idiopathic absence epilepsy. Epilepsy research. 2000;39(1):57-61. Epub 2000/02/26. PubMed PMID: 10690754.

63. Wilkie H, Osei-Lah A, Chioza B, Nashef L, McCormick D, Asherson P, et al. Association of mu-opioid receptor subunit gene and idiopathic generalized epilepsy. Neurology. 2002;59(5):724-8. Epub 2002/09/11. PubMed PMID: 12221164.

64. Durner M, Zhou G, Fu D, Abreu P, Shinnar S, Resor SR, et al. Evidence for linkage of adolescent-onset idiopathic generalized epilepsies to chromosome 8-and genetic heterogeneity. American journal of human genetics. 1999;64(5):1411-9. Epub 1999/04/17. doi: 10.1086/302371. PubMed PMID: 10205274; PubMed Central PMCID: PMCPMC1377879.

65. Rozycka A, Steinborn B, Trzeciak WH. The 1674+11C>T polymorphism of CHRNA4 is associated with juvenile myoclonic epilepsy. Seizure : the journal of the British Epilepsy Association. 2009;18(8):601-3. Epub 2009/07/07. doi: 10.1016/j.seizure.2009.06.007. PubMed PMID: 19577488.

66. Klassen T, Davis C, Goldman A, Burgess D, Chen T, Wheeler D, et al. Exome sequencing of ion channel genes reveals complex profiles confounding personal risk assessment in epilepsy. Cell. 2011;145(7):1036-48. Epub 2011/06/28. doi: 10.1016/j.cell.2011.05.025. PubMed PMID: 21703448; PubMed Central PMCID: PMCPMC3131217.

67. Lal D, Ruppert AK, Trucks H, Schulz H, de Kovel CG, Kasteleijn-Nolst Trenite D, et al. Burden analysis of rare microdeletions suggests a strong impact of neurodevelopmental genes in genetic generalised epilepsies. PLoS genetics. 2015;11(5):e1005226. Epub 2015/05/08. doi: 10.1371/journal.pgen.1005226. PubMed PMID: 25950944; PubMed Central PMCID: PMCPMC4423931.

68. Tang B, Sander T, Craven KB, Hempelmann A, Escayg A. Mutation analysis of the hyperpolarization-activated cyclic nucleotide-gated channels HCN1 and HCN2 in idiopathic generalized epilepsy. Neurobiology of disease. 2008;29(1):59-70. Epub 2007/10/13. doi: 10.1016/j.nbd.2007.08.006. PubMed PMID: 17931874; PubMed Central PMCID: PMCPMC2709210.

69. Galizia EC, Myers CT, Leu C, de Kovel CG, Afrikanova T, Cordero-Maldonado ML, et al. CHD2 variants are a risk factor for photosensitivity in epilepsy. Brain : a journal of neurology. 2015;138(Pt 5):1198-207. Epub 2015/03/19. doi: 10.1093/brain/awv052. PubMed PMID: 25783594; PubMed Central PMCID: PMCPMC4407192.

70. Phani NM, Acharya S, Xavy S, Bhaskaranand N, Bhat MK, Jain A, et al. Genetic association of KCNJ10 rs1130183 with seizure susceptibility and computational analysis of deleterious non-synonymous SNPs of KCNJ10 gene. Gene. 2014;536(2):247-53. Epub 2014/01/01. doi: 10.1016/j.gene.2013.12.026. PubMed PMID: 24378235.

71. Buono RJ, Lohoff FW, Sander T, Sperling MR, O'Connor MJ, Dlugos DJ, et al. Association between variation in the human KCNJ10 potassium ion channel gene and seizure susceptibility. Epilepsy research. 2004;58(2-3):175-83. Epub 2004/05/04. doi: 10.1016/j.eplepsyres.2004.02.003. PubMed PMID: 15120748.

72. Lenzen KP, Heils A, Lorenz S, Hempelmann A, Hofels S, Lohoff FW, et al. Supportive evidence for an allelic association of the human KCNJ10 potassium channel gene with idiopathic generalized epilepsy. Epilepsy research. 2005;63(2-3):113-8. Epub 2005/02/24. doi: 10.1016/j.eplepsyres.2005.01.002. PubMed PMID: 15725393.

73. Sapio MR, Vessaz M, Thomas P, Genton P, Fricker LD, Salzmann A. Novel carboxypeptidase A6 (CPA6) mutations identified in patients with juvenile myoclonic and generalized epilepsy. PloS one. 2015;10(4):e0123180. Epub 2015/04/16. doi: 10.1371/journal.pone.0123180. PubMed PMID: 25875328; PubMed Central PMCID: PMCPMC4395397.
