# Supplementary material for: Multi-gene panel testing in Korean patients with common genetic generalized epilepsy syndromes
Source: PLoS One. 2018 Jun 20;13(6):e0199321. doi: 10.1371/journal.pone.0199321 (PMC6010271; doi:10.1371/journal.pone.0199321)
Supplement: S2 Table — (DOCX) [file pone.0199321.s002.docx]

**S2 Table.** Characteristics of the identified 3 pathogenic and 19 likely pathogenic variants according to the American College of Medical Genetics and Genomics (ACMG) classification in the 57 patients.

| N | Gene | Isoform | Chr | Exome | HGVS.c | HGVS.p | Zygosity | AF  in 57 | 1000Gp3_AF | ExAC_AF | 1100  _AF | P-value (1100) | CLINVAR | HGMD | Prediction | | ACMG Criteria | | ACMG Classification | |  |
| --- | --- | --- | --- | --- | --- | --- | --- | --- | --- | --- | --- | --- | --- | --- | --- | --- | --- | --- | --- | --- | --- |
| 1 | *GABRD* | NM_000815.4 | chr1 | 6/9 | c.688T>G | p.(Ser230Ala) | Het | 0.01 | . | . | . | 0.049 | . | . | 3/10 | | PS4,PM2,BP4 | | LP | |  |
| 2 | *SZT2* | NM_015284.3 | chr1 | 27/71 | c.3973G>T | p.(Ala1325Ser) | Het | 0.01 | . | . | . | 0.049 | . | . | 1/9 | | PS4,PM2,BP4 | | LP | |  |
| 3 | *SZT2* | NM_015284.3 | chr1 | 68/71 | c.9797T>C | p.(Leu3266Pro) | Het | 0.01 | . | . | . | 0.049 | . | . | 6/9 | | PS4,PM2,PP3 | | LP | |  |
| 4 | *DOCK7* | NM_001271999.1 | chr1 | 30/49 | c.3647T>C | p.(Val1216Ala) | Het | 0.01 | . | . | . | 0.049 | . | . | 4/10 | | PS4,PM2BP4 | | LP | |  |
| 5 | *SCN1A* | NM_001165963.1 | chr2 | 22/26 | c.4319C>G | p.(Ala1440Gly) | Het | 0.01 | . | . | . | 0.049 | . | . | 10/10 | | PS4,PM2,PP3 | | LP | |  |
| 6 | *GPR98* | NM_032119.3 | chr5 | 45/90 | c.9679C>T | p.(Arg3227*) | Het | 0.01 | . | 0.0000 | . | 0.049 | . | . | NA | | PSV1,PS4 | | P | |  |
| 7 | *GRM4* | NM_000841.3 | chr6 | 7/11 | c.1360A>T | p.(Asn454Tyr) | Het | 0.01 | . | . | . | 0.049 | . | . | 9/10 | | PS4,PM2,PP3 | | LP | |  |
| 8 | *GRM4* | NM_001256809.2 | chr6 | 1/9 | c.88C>T | p.(Pro30Ser) | Het | 0.01 | . | . | . | 0.049 | . | . | 2/6 | | PS4,PM2,BP4 | | LP | |  |
| 9 | *CPA6* | NM_020361.4 | chr8 | 1/11 | c.54delC | p.(Trp19Glyfs*4) | Het | 0.01 | . | . | . | 0.049 | . | . | NA | | PSV1,PS4,PM2 | | P | |  |
| 10 | *CHRNB3* | NM_000749.3 | chr8 | 6/6 | c.1373_1374insCATC | p.(*459Ileext*4) | Het | 0.01 | . | . | . | 0.049 | . | . | NA | | PS4,PM2 | | LP | |  |
| 11 | *GATM* | NM_001482.2 | chr15 | 5/9 | c.790G>A | p.(Asp264Asn) | Het | 0.01 | . | . | . | 0.049 | . | . | 7/10 | | PS4,PM2,PP3 | | LP | |  |
| 12 | *POLG* | NM_001126131.1 | chr15 | 3/23 | c.752C>G | p.(Thr251Ser) | Het | 0.01 | . | . | . | 0.049 | . | . | 1/10 | | PS4,PM2,BP4 | | LP | |  |
| 13 | *CACNA1H* | NM_021098.2 | chr16 | 2/35 | c.175G>T | p.(Gly59Cys) | Het | 0.01 | . | . | . | 0.049 | . | . | 6/10 | | PS4,PM2,PP3 | | LP | |  |
| 14 | *CACNA1H* | NM_021098.2 | chr16 | 10/35 | c.2363G>A | p.(Arg788His) | Het | 0.01 | 0.0002 | 0.0000 | . | 0.049 | . | . | 8/10 | | PS4,PM5,PP3,PP1 | | LP | |  |
| 15 | *CACNA1H* | NM_021098.2 | chr16 | 35/35 | c.6586C>A | p.(Pro2196Thr) | Het | 0.01 | . | . | . | 0.049 | . | . | 5/10 | | PS4,PM2 | | LP | |  |
| 16 | *CACNA1H* | NM_021098.2 | chr16 | 35/35 | c.7004A>G | p.(Lys2335Arg) | Het | 0.01 | . | . | . | 0.049 | . | . | 6/10 | | PS4,PM2,PP3 | | LP | |  |
| 17 | *NDE1* | NM_001143979.1 | chr16 | 6/10 | c.457G>A | p.(Glu153Lys) | Het | 0.01 | . | . | . | 0.049 | . | . | 9/9 | | PS4,PM2,PP3 | | LP | |  |
| 18 | *CACNA1G* | NM_018896.4 | chr17 | 38/38 | c.6496G>A | p.(Ala2166Thr) | Het | 0.01 | . | . | . | 0.049 | . | . | 2/10 | | PS4,PM2BP4 | | LP | |  |
| 19 | *CACNA1A* | NM_023035.2 | chr19 | 5/47 | c.714delT | p.(Ile239Phefs*5) | Het | 0.01 | . | . | . | 0.084 | 0.049 | . | | . | NA | PSV1,PS4,PM2 | | P | |
| 20 | *CACNA1A* | NM_023035.2 | chr19 | 18/47 | c.2204A>C | p.(Glu735Ala) | Het | 0.01 | 0.0040 | 0.0101 | . | 0.084 | 0.049 | LB | | . | 7/10 | PS4,PP3,BS1, | | LP | |
| 21 | *LGI4* | NM_139284.2 | chr19 | 8/9 | c.1123C>T | p.(Arg375Trp) | Het | 0.01 | . | . | . | 0.084 | 0.049 | . | | . | 5/10 | PS4,PM2 | | LP | |
| 22 | *ARX* | NM_139058.2 | chrX | 2/5 | c.847G>A | p.(Ala283Thr) | Het | 0.01 | . | . | . | 0.084 | 0.049 | . | | . | 2/10 | PS4,PM2,BP4 | | LP | |

ACMG criteria: PVS1, null variant in a gene where loss of function is a known mechanism of disease; PS4, prevalence of the variant in affected individuals is significantly increased compared with the prevalence in controls; PM2, absent from control in population database; PM5, novel missense change at an amino acid residue where a different missense change determined to be pathogenic has been seen before; PP1, cosegregation with disease in affected family members; PP3, multiple lines of computational evidence suggest a deleterious effect on the

gene or gene product; BS1, allele frequency is greater than expected for disorder; BP4, multiple lines of computational evidence suggest no impact on gene or gene product

N: number; Chr: chromosome; AF: allele frequency; Het: heterozygous; LB: likely benign; LP: likely pathogenic; P: pathogenic.
