# Supplementary material for: Multi-gene panel testing in Korean patients with common genetic generalized epilepsy syndromes
Source: PLoS One. 2018 Jun 20;13(6):e0199321. doi: 10.1371/journal.pone.0199321 (PMC6010271; doi:10.1371/journal.pone.0199321)
Supplement: S3 Table — (DOCX) [file pone.0199321.s003.docx]

**S3 Table.** Characteristics of the isolated 109 variants of uncertain significance according to American College of Medical Genetics and Genomics (ACMG) classification in the 57 patients.

| N | Gene | Isoform | Chr | Exome | HGVS.c | HGVS.p | Zygosity | AF in 57 | 1000Gp3_AF | ExAC_AF | 1100  _AF | *p*-value (1100) | CLINVAR | HGMD | Prediction | ACMG Criteria | ACMG Classification |
| --- | --- | --- | --- | --- | --- | --- | --- | --- | --- | --- | --- | --- | --- | --- | --- | --- | --- |
| 1 | *GABRD* | NM_000815.4 | chr1 | 9/9 | c.1202C>T | p.(Thr401Met) | Het | 0.01 | 0.0086 | 0.0020 | 0.0027 | 0.255 | . | . | 2/10 | BP4 | US |
| 2 | *SZT2* | NM_015284.3 | chr1 | 25/71 | c.3467G>A | p.(Arg1156His) | Het | 0.01 | 0.0004 | 0.0003 | 0.0023 | 0.230 | . | . | 0/9 | BP4 | US |
| 3 | *GJA8* | NM_005267.4 | chr1 | 2/2 | c.356G>A | p.(Gly119Glu) | Het | 0.01 | 0.0002 | 0.0001 | 0.0059 | 0.306 | . | . | 1/9 | BP4 | US |
| 4 | *CHRNB2* | NM_000748.2 | chr1 | 5/6 | c.1190A>C | p.(Gln397Pro) | Het | 0.01 | . | . | 0.0005 | 0.096 | . | . | 1/10 | BP4 | US |
| 5 | *NRXN1* | NM_001135659.1 | chr2 | 22/24 | c.4180A>T | p.(Thr1394Ser) | Het | 0.01 | 0.0008 | 0.0002 | 0.0027 | 0.255 | . | . | 3/10 | BP4 | US |
| 6 | *NRXN1* | NM_001135659.1 | chr2 | 20/24 | c.3760G>A | p.(Val1254Ile) | Het | 0.01 | . | 0.0000 | 0.0018 | 0.202 | . | . | 1/10 | BP4 | US |
| 7 | *NRXN1* | NM_001135659.1 | chr2 | 19/24 | c.3523A>G | p.(Ile1175Val) | Het | 0.01 | . | 0.0001 | 0.0014 | 0.170 | . | . | 2/10 | BP4 | US |
| 8 | *NRXN1* | NM_001135659.1 | chr2 | 11/24 | c.2090G>A | p.(Arg697Gln) | Het | 0.01 | . | 0.0000 | 0.0005 | 0.096 | . | . | 4/10 | BP4 | US |
| 9 | *NRXN1* | NM_001135659.1 | chr2 | 2/24 | c.611T>A | p.(Leu204Gln) | Het | 0.01 | . | 0.0000 | . | 0.049 | . | . | 3/10 | PS4,BP4 | US |
| 10 | *ZEB2* | NM_014795.3 | chr2 | 8/10 | c.2004G>T | p.(Glu668Asp) | Het | 0.01 | . | 0.0002 | 0.0041 | 0.314 | . | . | 8/10 | PP3 | US |
| 11 | *CACNB4* | NM_000726.3 | chr2 | 2/14 | c.86G>A | p.(Arg29Gln) | Het | 0.01 | . | 0.0000 | . | 0.049 | . | . | 5/10 | PS4 | US |
| 12 | *SCN3A* | NM_006922.3 | chr2 | 28/28 | c.5843A>C | p.(Lys1948Thr) | Het | 0.01 | 0.0002 | 0.0001 | 0.0027 | 0.255 | . | . | 8/10 | PP3 | US |
| 13 | *SCN3A* | NM_006922.3 | chr2 | 28/28 | c.5407G>A | p.(Asp1803Asn) | Het | 0.01 | 0.0026 | 0.0014 | 0.0177 | 0.405 | . | . | 6/10 | PP3,BS1 | US |
| 14 | *SCN3A* | NM_006922.3 | chr2 | 12/28 | c.1602A>C | p.(Arg534Ser) | Het | 0.03 | 0.0014 | 0.0001 | 0.0077 | 0.057 | . | . | 10/10 | PS4,PP3 | US |
| 15 | *SCN2A* | NM_001040142.1 | chr2 | 8/27 | c.982T>G | p.(Phe328Val) | Het | 0.01 | . | 0.0001 | 0.0045 | 0.328 | . | . | 4/10 | BP4 | US |
| 16 | *SCN1A* | NM_001165963.1 | chr2 | 25/26 | c.4723C>T | p.(Arg1575Cys) | Het | 0.01 | 0.0004 | 0.0001 | 0.0045 | 0.328 | US | DM RE | 7/10 | PP3 | US |
| 17 | *SCN9A* | NM_002977.3 | chr2 | 27/27 | c.5678G>A | p.(Arg1893His) | Het | 0.01 | 0.0014 | 0.0003 | 0.0055 | 0.351 | . | . | 9/10 | PP3 | US |
| 18 | *SCN9A* | NM_002977.3 | chr2 | 15/27 | c.2359A>G | p.(Met787Val) | Het | 0.04 | 0.0046 | 0.0019 | 0.0295 | 0.523 | . | . | 7/10 | PP3,BS1 | US |
| 19 | *SCN9A* | NM_002977.3 | chr2 | 12/27 | c.1818T>G | p.(Ser606Arg) | Het | 0.01 | 0.0002 | 0.0001 | 0.0027 | 0.255 | . | . | 9/10 | PP3 | US |
| 20 | *CACNA2D2* | NM_001174051.2 | chr3 | 25/39 | c.2116A>G | p.(Lys706Glu) | Het | 0.01 | . | 0.0001 | 0.0009 | 0.134 | . | . | 0/10 | BP4 | US |
| 21 | *CASR* | NM_001178065.1 | chr3 | 7/7 | c.1805A>G | p.(Asn602Ser) | Het | 0.01 | 0.0094 | 0.0033 | 0.0186 | 0.378 | . | . | 6/10 | PP3,BS1, | US |
| 22 | *CASR* | NM_001178065.1 | chr3 | 7/7 | c.2854G>A | p.(Glu952Lys) | Het | 0.01 | 0.0018 | . | 0.0055 | 0.351 | . | . | 1/10 | BP4 | US |
| 23 | *CLCN2* | NM_004366.5 | chr3 | 17/24 | c.1991A>T | p.(Glu664Val) | Het | 0.01 | 0.0030 | 0.0006 | 0.0059 | 0.359 | . | . | 3/10 | BP4 | US |
| 24 | *CLCN2* | NM_004366.5 | chr3 | 15/24 | c.1705G>A | p.(Gly569Ser) | Het | 0.01 | . | 0.0000 | 0.0005 | 0.096 | . | . | 8/10 | PP3 | US |
| 25 | *LIAS* | NM_001278591.1 | chr4 | 4/4 | c.403G>A | p.(Ala135Thr) | Het | 0.02 | . | . | 0.0095 | 0.214 | B | . | 0/0 | BP4 | US |
| 26 | *LIAS* | NM_006859.3 | chr4 | 8/11 | c.790C>T | p.(His264Tyr) | Het | 0.01 | . | . | 0.0009 | 0.134 | . | . | 3/10 | BP4 | US |
| 27 | *MFSD8* | NM_152778.2 | chr4 | 5/13 | c.206C>T | p.(Pro69Leu) | Het | 0.01 | 0.0004 | 0.0003 | 0.0077 | 0.343 | . | . | 4/10 | BP4 | US |
| 28 | *GPR98* | NM_032119.3 | chr5 | 12/90 | c.2330A>C | p.(Glu777Ala) | Het | 0.01 | 0.0010 | 0.0001 | 0.0032 | 0.277 | . | . | 7/10 | PP3 | US |
| 29 | *GPR98* | NM_032119.3 | chr5 | 21/90 | c.4703G>A | p.(Ser1568Asn) | Het | 0.01 | . | 0.0001 | 0.0009 | 0.134 | . | DM D | 4/10 | BP4 | US |
| 30 | *GPR98* | NM_032119.3 | chr5 | 28/90 | c.5771T>C | p.(Ile1924Thr) | Het | 0.01 | . | 0.0000 | . | 0.049 | . | . | 1/10 | PS4,BP4 | US |
| 31 | *GPR98* | NM_032119.3 | chr5 | 30/90 | c.6559A>G | p.(Ile2187Val) | Het | 0.01 | 0.0014 | 0.0009 | 0.0077 | 0.377 | LB | DM D | 2/10 | BP4 | US |
| 32 | *GPR98* | NM_032119.3 | chr5 | 63/90 | c.12704A>G | p.(Tyr4235Cys) | Het | 0.01 | 0.0002 | 0.0002 | . | 0.049 | . | DM D | 7/10 | PS4,PP3 | US |
| 33 | *GPR98* | NM_032119.3 | chr5 | 66/90 | c.13340G>A | p.(Gly4447Asp) | Het | 0.01 | . | 0.0001 | 0.0014 | 0.170 | . | . | 6/10 | PP3 | US |
| 34 | *GPR98* | NM_032119.3 | chr5 | 69/90 | c.13996A>G | p.(Ile4666Val) | Het | 0.01 | . | 0.0001 | 0.0073 | 0.375 | . | DM D | 1/10 | BP4 | US |
| 35 | *GPR98* | NM_032119.3 | chr5 | 70/90 | c.14515C>G | p.(Gln4839Glu) | Het | 0.03 | 0.0064 | 0.0019 | 0.0355 | 0.427 | B | DM D | 6/10 | PP3,BS1, | US |
| 36 | *GPR98* | NM_032119.3 | chr5 | 74/90 | c.15293A>C | p.(Glu5098Ala) | Het | 0.01 | . | 0.0000 | . | 0.049 | . | . | 6/10 | PS4,PP3 | US |
| 37 | *GPR98* | NM_032119.3 | chr5 | 77/90 | c.16439G>A | p.(Ser5480Asn) | Het | 0.01 | . | 0.0001 | 0.0023 | 0.230 | . | . | 6/10 | PP3 | US |
| 38 | *GPR98* | NM_032119.3 | chr5 | 77/90 | c.16537G>A | p.(Ala5513Thr) | Het | 0.01 | . | 0.0000 | 0.0009 | 0.134 | . | . | 0/10 | BP4 | US |
| 39 | *GPR98* | NM_032119.3 | chr5 | 79/90 | c.17033G>C | p.(Gly5678Ala) | Het | 0.01 | . | 0.0001 | 0.0036 | 0.297 | . | . | 2/10 | BP4 | US |
| 40 | *GPR98* | NM_032119.3 | chr5 | 88/90 | c.18601A>C | p.(Asn6201His) | Het | 0.01 | 0.0004 | 0.0001 | . | 0.049 | . | . | 6/10 | PS4,PP3 | US |
| 41 | *ALDH7A1* | NM_001182.4 | chr5 | 12/18 | c.1016A>G | p.(His339Arg) | Het | 0.02 | 0.0002 | 0.0002 | 0.0041 | 0.085 | . | . | 9/10 | PP3 | US |
| 42 | *ALDH7A1* | NM_001202404.1 | chr5 | 1/16 | c.63C>G | p.(Ile21Met) | Het | 0.01 | . | . | . | 0.049 | . | . | 1/9 | PS4,BP4 | US |
| 43 | *GABRG2* | NM_198903.2 | chr5 | 1/11 | c.24C>A | p.(Ser8Arg) | Het | 0.01 | 0.0002 | 0.0000 | 0.0005 | 0.096 | . | . | 1/10 | BP4 | US |
| 44 | *GRM4* | NM_000841.3 | chr6 | 10/11 | c.2560C>A | p.(Pro854Thr) | Het | 0.01 | . | 0.0000 | 0.0018 | 0.202 | . | . | 3/10 | BP4 | US |
| 45 | *GRM4* | NM_000841.3 | chr6 | 6/11 | c.1052G>A | p.(Arg351His) | Het | 0.01 | . | 0.0000 | 0.0014 | 0.170 | . | . | 10/10 | PP3 | US |
| 46 | *EPM2A* | NM_005670.3 | chr6 | 1/4 | c.136G>C | p.(Ala46Pro) | Het | 0.10 | 0.0116 | 0.0068 | 0.0377 | 0.003 | . | . | 4/10 | PS4,BS1,BP4 | US |
| 47 | *EFHC1* | NM_018100.3 | chr6 | 9/11 | c.1523C>G | p.(Thr508Arg) | Het | 0.01 | . | 0.0001 | 0.0036 | 0.297 | . | DM JME | 1/10 | PS1,BP4 | US |
| 48 | *OPRM1* | NM_001145279.3 | chr6 | 5/6 | c.1156G>A | p.(Val386Ile) | Het | 0.01 | 0.0026 | 0.0008 | 0.0027 | 0.255 | . | . | 0/10 | BP4 | US |
| 49 | *OPRM1* | NM_001008504.3 | chr6 | 3/3 | c.1168C>T | p.(Arg390Cys) | Het | 0.02 | 0.0002 | 0.0002 | 0.0027 | 0.050 | . | . | 2/8 | PS4,BP4 | US |
| 50 | *CNTNAP2* | NM_014141.5 | chr7 | 10/24 | c.1620G>C | p.(Lys540Asn) | Het | 0.01 | . | 0.0000 | . | 0.034 | . | . | 1/10 | PS4,BP4 | US |
| 51 | *CHRNA2* | NM_000742.3 | chr8 | 6/7 | c.1144C>T | p.(Arg382Trp) | Het | 0.01 | 0.0002 | 0.0000 | 0.0005 | 0.096 | . | . | 10/10 | PP3 | US |
| 52 | *CHRNA2* | NM_000742.3 | chr8 | 6/7 | c.1073G>T | p.(Ser358Ile) | Het | 0.01 | . | 0.0001 | . | 0.049 | . | . | 9/10 | PS4,PP3 | US |
| 53 | *CHRNB3* | NM_000749.3 | chr8 | 2/6 | c.130G>T | p.(Val44Phe) | Het | 0.02 | . | 0.0000 | . | 0.002 | . | . | 7/10 | PS4,PP3 | US |
| 54 | *KCNQ3* | NM_004519.3 | chr8 | 15/15 | c.1918G>A | p.(Val640Met) | Het | 0.01 | . | 0.0000 | . | 0.049 | . | . | 8/10 | PS4,PP3 | US |
| 55 | *JRK* | NM_003724.3 | chr8 | 3/3 | c.1561G>A | p.(Val521Met) | Het | 0.01 | . | . | . | 0.049 | . | . | 0/0 | PS4,PM2,BP4 | US |
| 56 | *JRK* | NM_003724.3 | chr8 | 2/3 | c.1301A>G | p.(Gln434Arg) | Het | 0.01 | . | . | 0.0036 | 0.297 | . | . | 0/0 | BP4 | US |
| 57 | *JRK* | NM_003724.3 | chr8 | 2/3 | c.1163C>T | p.(Ser388Leu) | Het | 0.01 | 0.0002 | . | . | 0.049 | . | . | 0/0 | PS4,BP4 | US |
| 58 | *JRK* | NM_003724.3 | chr8 | 2/3 | c.1084G>A | p.(Ala362Thr) | Het | 0.01 | 0.0004 | . | 0.0036 | 0.297 | . | . | 0/0 | BP4 | US |
| 59 | *STXBP1* | NM_003165.3 | chr9 | 15/20 | c.1281C>G | p.(Ile427Met) | Het | 0.01 | . | . | 0.0014 | 0.170 | . | . | 3/10 | BP4 | US |
| 60 | *SPTAN1* | NM_001130438.2 | chr9 | 22/57 | c.3101A>G | p.(Asn1034Ser) | Het | 0.01 | . | 0.0000 | 0.0005 | 0.096 | . | . | 5/10 |  | US |
| 61 | *SPTAN1* | NM_001130438.2 | chr9 | 47/57 | c.6014A>G | p.(Lys2005Arg) | Het | 0.01 | . | 0.0000 | 0.0014 | 0.170 | . | . | 4/10 | BP4 | US |
| 62 | *KCNMA1* | NM_001271522.1 | chr10 | 2/2 | c.400G>C | p.(Glu134Gln) | Het | 0.03 | 0.0156 | . | . | 0.000 | . | . | 0/0 | PS4,BS1,BP4 | US |
| 63 | *LGI1* | NM_005097.2 | chr10 | 8/8 | c.1220G>A | p.(Arg407His) | Het | 0.01 | 0.0004 | 0.0001 | . | 0.049 | . | . | 5/10 | PS4 | US |
| 64 | *SLC25A22* | NM_001191060.1 | chr11 | 4/10 | c.151G>A | p.(Asp51Asn) | Het | 0.01 | 0.0030 | 0.0006 | 0.0005 | 0.096 | B/US | . | 8/10 | PP3 | US |
| 65 | *TPP1* | NM_000391.3 | chr11 | 8/13 | c.1049G>A | p.(Arg350Gln) | Het | 0.01 | 0.0004 | 0.0000 | 0.0005 | 0.067 | . | . | 9/10 | PP3 | US |
| 66 | *FOLR1* | NM_000802.3 | chr11 | 3/5 | c.292C>T | p.(Arg98Trp) | Het | 0.01 | 0.0014 | 0.0033 | 0.0041 | 0.314 | . |  | 3/10 | BP4 | US |
| 67 | *PRICKLE1* | NM_001144881.1 | chr12 | 8/8 | c.2194G>A | p.(Gly732Arg) | Het | 0.01 | 0.0010 | 0.0002 | 0.0027 | 0.255 | . | . | 1/10 | BP4 | US |
| 68 | *PRICKLE1* | NM_001144881.1 | chr12 | 7/8 | c.1621G>T | p.(Ala541Ser) | Het | 0.01 | . | 0.0000 | 0.0018 | 0.202 | . | . | 6/10 | PP3 | US |
| 69 | *PRICKLE1* | NM_001144881.1 | chr12 | 2/8 | c.113C>T | p.(Pro38Leu) | Het | 0.01 | 0.0006 | 0.0004 | 0.0068 | 0.371 | . | . | 10/10 | PP3 | US |
| 70 | *SCN8A* | NM_014191.3 | chr12 | 7/27 | c.875A>G | p.(Tyr292Cys) | Het | 0.01 | . | 0.0000 | 0.0005 | 0.096 | . | . | 7/10 | PP3 | US |
| 71 | *UBE3A* | NM_000462.3 | chr15 | 7/14 | c.836G>A | p.(Arg279Gln) | Het | 0.01 | . | 0.0000 | 0.0005 | 0.096 | . | . | 2/10 | BP4 | US |
| 72 | *UBE3A* | NM_000462.3 | chr15 | 7/14 | c.601G>A | p.(Ala201Thr) | Het | 0.01 | 0.0034 | 0.0123 | . | 0.049 | B/US | . | 1/10 | PS4,BS1,BP4 | US |
| 73 | *GABRB3* | NM_021912.4 | chr15 | 1/9 | c.17T>C | p.(Leu6Pro) | Het | 0.01 | . | 0.0000 | . | 0.049 | . | . | 1/9 | PS4,BP4 | US |
| 74 | *CHRNA7* | NM_001190455.2 | chr15 | 5/10 | c.457G>A | p.(Ala153Thr) | Het | 0.03 | . | 0.0013 | 0.0036 | 0.030 | . | . | 6/10 | PS4,PP3 | US |
| 75 | *CHD2* | NM_001271.3 | chr15 | 32/39 | c.4036G>T | p.(Val1346Leu) | Het | 0.01 | 0.0002 | 0.0000 | . | 0.049 | . | . | 3/10 | PS4,BP4 | US |
| 76 | *CHD2* | NM_001271.3 | chr15 | 35/39 | c.4472A>G | p.(Lys1491Arg) | Het | 0.01 | . | . | 0.0005 | 0.096 | . | . | 3/10 | BP4 | US |
| 77 | *CHD2* | NM_001271.3 | chr15 | 37/39 | c.4874A>G | p.(Asn1625Ser) | Het | 0.01 | . | 0.0000 | . | 0.049 | . | . | 1/10 | PS4,BP4 | US |
| 78 | *CACNA1H* | NM_021098.2 | chr16 | 27/35 | c.4780G>T | p.(Ala1594Ser) | Het | 0.01 | 0.0002 | 0.0001 | 0.0036 | 0.297 | . | . | 10/10 | PP3 | US |
| 79 | *CACNA1H* | NM_021098.2 | chr16 | 27/35 | c.4781C>T | p.(Ala1594Val) | Het | 0.01 | 0.0002 | 0.0001 | 0.0041 | 0.314 | . | . | 10/10 | PP3 | US |
| 80 | *CACNA1H* | NM_021098.2 | chr16 | 33/35 | c.5479C>T | p.(His1827Tyr) | Het | 0.01 | 0.0010 | 0.0001 | 0.0009 | 0.134 | . | . | 8/10 | PP3 | US |
| 81 | *TBC1D24* | NM_001199107.1 | chr16 | 2/8 | c.22T>C | p.(Cys8Arg) | Het | 0.02 | 0.0028 | 0.0006 | 0.0082 | 0.186 | . | . | 2/10 | BP4 | US |
| 82 | *TBC1D24* | NM_001199107.1 | chr16 | 2/8 | c.601G>A | p.(Val201Met) | Het | 0.01 | 0.0002 | 0.0000 | 0.0009 | 0.134 | . | . | 3/10 | BP4 | US |
| 83 | *GRIN2A* | NM_000833.4 | chr16 | 4/14 | c.559G>A | p.(Val187Ile) | Het | 0.01 | . | 0.0001 | 0.0005 | 0.096 | . | . | 1/10 | BP4 | US |
| 84 | *NDE1* | NM_001143979.1 | chr16 | 7/10 | c.604A>G | p.(Thr202Ala) | Het | 0.01 | 0.0002 | 0.0000 | 0.0005 | 0.096 | . | . | 3/9 | BP4 | US |
| 85 | *PRRT2* | NM_001256442.1 | chr16 | 2/3 | c.621A>C | p.(Lys207Asn) | Het | 0.01 | . | . | . | 0.049 | . | . | 0/10 | PS4,BP4 | US |
| 86 | *SPECC1* | NM_001033553.2 | chr17 | 2/15 | c.52G>A | p.(Gly18Ser) | Het | 0.01 | . | 0.0000 | 0.0014 | 0.124 | . | . | 5/10 |  | US |
| 87 | *SPECC1* | NM_001033553.2 | chr17 | 4/15 | c.701A>G | p.(Lys234Arg) | Het | 0.01 | . | 0.0000 | . | 0.034 | . | . | 1/10 | PS4,BP4 | US |
| 88 | *CACNA1G* | NM_018896.4 | chr17 | 9/38 | c.2020G>A | p.(Gly674Arg) | Het | 0.01 | 0.0002 | 0.0002 | 0.0041 | 0.314 | . | . | 5/10 |  | US |
| 89 | *CACNA1G* | NM_018896.4 | chr17 | 17/38 | c.3295G>A | p.(Ala1099Thr) | Het | 0.01 | 0.0054 | 0.0009 | 0.0023 | 0.230 | . | . | 4/10 | BP4 | US |
| 90 | *CACNA1G* | NM_018896.4 | chr17 | 18/38 | c.3728G>A | p.(Arg1243Gln) | Het | 0.01 | 0.0010 | 0.0001 | 0.0023 | 0.230 | . | . | 5/10 |  | US |
| 91 | *ME2* | NM_001168335.1 | chr18 | 14/14 | c.1432A>G | p.(Ile478Val) | Het | 0.04 | 0.0026 | 0.0016 | 0.0064 | 0.009 | . | . | 1/8 | PS4,BS1,BP4 | US |
| 92 | *CACNA1A* | NM_023035.2 | chr19 | 20/47 | c.3248C>A | p.(Ala1083Asp) | Het | 0.01 | . | . | 0.0005 | 0.096 | . | . | 2/10 | BP4 | US |
| 93 | *CACNA1A* | NM_023035.2 | chr19 | 20/47 | c.3178C>T | p.(Arg1060Cys) | Het/Hom | 0.03 | 0.0008 | 0.0003 | 0.0136 | 0.144 | . | . | 8/10 | PP3,BS1, | US |
| 94 | *CACNA1A* | NM_023035.2 | chr19 | 8/47 | c.1168A>G | p.(Asn390Asp) | Het | 0.01 | . | . | 0.0005 | 0.096 | . | . | 10/10 | PP3 | US |
| 95 | *SCN1B* | NM_199037.3 | chr19 | 3/3 | c.785G>A | p.(Cys262Tyr) | Het | 0.01 | . | 0.0000 | 0.0005 | 0.096 | . | . | 3/8 | BP4 | US |
| 96 | *SCN1B* | NM_001037.4 | chr19 | 4/6 | c.566C>T | p.(Thr189Met) | Het | 0.01 | 0.0006 | 0.0002 | 0.0041 | 0.314 | . | DM AF | 8/10 | PP3 | US |
| 97 | *LGI4* | NM_139284.2 | chr19 | 7/9 | c.766C>T | p.(Arg256Cys) | Het | 0.01 | . | 0.0001 | 0.0014 | 0.170 | . | . | 3/10 | BP4 | US |
| 98 | *PNKP* | NM_007254.3 | chr19 | 11/17 | c.1009G>C | p.(Glu337Gln) | Het | 0.01 | . | 0.0000 | 0.0005 | 0.096 | . | . | 1/10 | BP4 | US |
| 99 | *PNKP* | NM_007254.3 | chr19 | 7/17 | c.670C>T | p.(Arg224Cys) | Het | 0.01 | . | 0.0000 | . | 0.049 | . | . | 9/10 | PS4,PP3 | US |
| 100 | *PNKP* | NM_007254.3 | chr19 | 2/17 | c.56C>T | p.(Ala19Val) | Het | 0.01 | 0.0008 | 0.0000 | 0.0009 | 0.134 | . | DM ASD | 0/10 | BP4 | US |
| 101 | *CHRNA4* | NM_000744.6 | chr20 | 6/6 | c.1841C>T | p.(Thr614Met) | Het | 0.01 | . | 0.0000 | . | 0.034 | . | . | 10/10 | PS4,PP3 | US |
| 102 | *CHRNA4* | NM_000744.6 | chr20 | 5/6 | c.1459C>T | p.(Arg487Trp) | Het | 0.01 | 0.0004 | 0.0001 | 0.0041 | 0.251 | . | . | 1/10 | BP4 | US |
| 103 | *KCNQ2* | NM_172107.2 | chr20 | 17/17 | c.2264A>G | p.(Tyr755Cys) | Het | 0.01 | 0.0016 | 0.0011 | 0.0059 | 0.359 | LB | . | 7/10 | PP3 | US |
| 104 | *GRIK1* | NM_000830.3 | chr21 | 16/17 | c.2585G>A | p.(Arg862Gln) | Het | 0.01 | . | 0.0001 | 0.0009 | 0.134 | . | . | 6/10 | PP3 | US |
| 105 | *EFHC2* | NM_025184.3 | chrX | 10/15 | c.1457G>A | p.(Arg486His) | Het | 0.01 | 0.0021 | 0.0005 | 0.0123 | 1.000 | . | . | 7/10 | PP3,BS1 | US |
| 106 | *EFHC2* | NM_025184.3 | chrX | 9/15 | c.1408A>G | p.(Ile470Val) | Het | 0.01 | 0.0005 | 0.0003 | 0.0073 | 0.375 | . | . | 0/10 | BP4 | US |
| 107 | *EFHC2* | NM_025184.3 | chrX | 4/15 | c.394C>T | p.(Arg132Trp) | Het | 0.01 | 0.0008 | 0.0001 | 0.001 | 0.183 | . | . | 7/10 | PP3 | US |
| 108 | *PCDH19* | NM_001184880.1 | chrX | 6/6 | c.3400A>C | p.(Asn1134His) | Het | 0.04 | 0.0042 | 0.0013 | 0.015 | 0.036 | . | . | 0/10 | PS4,BS1,BP4 | US |
| 109 | *PCDH19* | NM_001184880.1 | chrX | 6/6 | c.3320G>A | p.(Arg1107His) | Het | 0.02 | 0.0003 | 0.0001 | 0.004 | 0.099 | . | . | 5/10 | BS1 | US |

ACMG criteria: PS4, prevalence of the variant in affected individuals is significantly increased compared with the prevalence in controls; PS1, Same amino acid change as a previously established pathogenic variant regardless of nucleotide change; PM2, absent from control in population database; PP3; multiple lines of computational evidence suggest a deleterious effect on the gene or gene product; BS1, allele frequency is greater than expected for disorder; BP4, multiple lines of computational evidence suggest no impact on gene or gene product.

N: number; Chr: chromosome; AF: allele frequency; Het: heterozygous; Hom: homogeneous; AF: allele frequency; LB: likely benign; US: uncertain significance; DM: disease causing mutation; RE: Rasmussen encephalitis; D: deafness; JME: juvenile myoclonic epilepsy; AF: atrial fibrillation; ASD: autism spectrum disorder.
